# Characterization of *Acinetobacter baumannii* core oligosaccharide synthesis reveals novel aspects of lipooligosaccharide assembly

**DOI:** 10.1128/mbio.03013-23

**Published:** 2024-02-13

**Authors:** Leah M. VanOtterloo, Luis A. Macias, Matthew J. Powers, Jennifer S. Brodbelt, M. Stephen Trent

**Affiliations:** 1 Department of Microbiology, College of Art and Sciences, University of Georgia, Athens, Georgia, USA; 2 Department of Chemistry, University of Texas at Austin, Austin, Texas, USA; 3 Department of Infectious Diseases, College of Veterinary Medicine, University of Georgia, Athens, Georgia, USA; The University of Kansas Medical Center, Kansas City, Kansas, USA

**Keywords:** lipooligosaccharide, LOS, lipopolysaccharide, LPS, Kdo, outer membrane, cell envelope, core oligosaccharide, lipid A

## Abstract

A fundamental feature of Gram-negative bacteria is their outer membrane that protects the cell against environmental stressors. This defense is predominantly due to its asymmetry, with glycerophospholipids located in the inner leaflet and lipopolysaccharide (LPS) or lipooligosaccharide (LOS) confined to the outer leaflet. LPS consists of a lipid A anchor, a core oligosaccharide, and a distal O-antigen while LOS lacks O-antigen. While LPS/LOS is typically essential for growth, this is not the case for *Acinetobacter baumannii*. Despite this unique property, the synthesis of the core oligosaccharide of *A. baumannii* LOS is not well-described. Here, we characterized the LOS chemotypes of *A. baumannii* strains with mutations in a predicted core oligosaccharide locus via tandem mass spectrometry. This allowed for an extensive identification of genes required for core assembly that can be exploited to generate precise structural LOS modifications in many *A. baumannii* strains. We further investigated two chemotypically identical yet phenotypically distinct mutants, ∆*2903* and ∆*lpsB*, that exposed a possible link between LOS and the peptidoglycan cell wall—two cell envelope components whose coordination has not yet been described in *A. baumannii*. Selective reconstruction of the core oligosaccharide via expression of 2903 and LpsB revealed that these proteins rely on each other for the unusual tandem transfer of two residues, KdoIII and N-acetylglucosaminuronic acid. The data presented not only allow for better usage of *A. baumannii* as a tool to study outer membrane integrity but also provide further evidence for a novel mechanism of core oligosaccharide assembly.

**IMPORTANCE:**

*Acinetobacter baumannii* is a multidrug-resistant pathogen that produces lipooligosaccharide (LOS), a glycolipid that confers protective asymmetry to the bacterial outer membrane. The core oligosaccharide is a ubiquitous component of LOS that typically follows a well-established model of synthesis. In addition to providing an extensive analysis of the genes involved in the synthesis of the core region, we demonstrate that this organism has evidently diverged from the long-held archetype of core synthesis. Moreover, our data suggest that *A. baumannii* LOS assembly is important for cell division and likely intersects with the synthesis of the peptidoglycan cell wall, another essential component of the Gram-negative cell envelope. This connection between LOS and cell wall synthesis provides an intriguing foundation for a unique method of outer membrane biogenesis and cell envelope coordination.

## INTRODUCTION

A central feature of Gram-negative bacteria is the outer membrane that acts as a barrier to protect the cell against a variety of toxic compounds (1, 2). This is achieved primarily by asymmetry of the outer membrane, with glycerophospholipids residing in the inner leaflet and lipopolysaccharide (LPS) confined to the outer leaflet (3, 4). LPS is a strongly negatively charged molecule made up of a lipid A anchor, a core oligosaccharide, and an O-antigen terminus (5). Many organisms possess a truncated chemotype of LPS termed lipooligosaccharide (LOS) which lacks the O-antigen component. Lipid A—a *bis*-phosphorylated and fatty-acylated glucosamine disaccharide—contributes primarily to outer membrane integrity through increased acyl chain packing and by cross-bridging with divalent cations, thereby promoting strong lateral interactions between adjacent LPS/LOS molecules and allowing for a selectively permeable membrane. Additional phosphate groups or electronegative sugar residues in the core oligosaccharide of many Gram-negative organisms can further contribute to an overall negative charge and provide more points for protective cross-bridging (6, 7).

**Fig 1:**
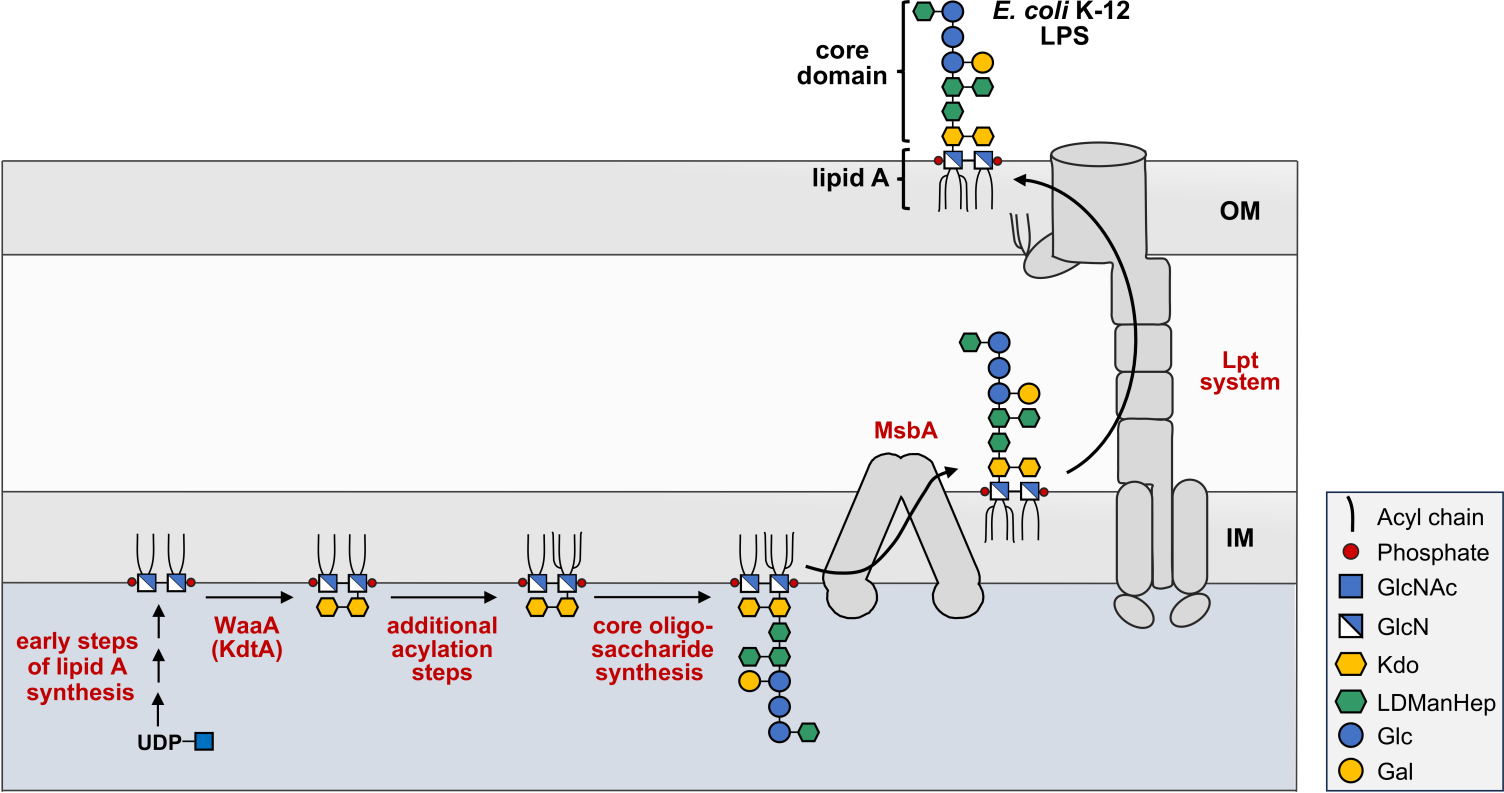
Model of LPS assembly and transport in *Escherichia coli* K-12. In the cytoplasmic-facing leaflet of the inner membrane (IM), early steps of the Raetz pathway of lipid A biosynthesis generate the lipid A precursor lipid IV_A_ from UDP-activated N-acetyl-glucosamine. Two 3-deoxy-D-*manno*-octulosonic acid (Kdo) sugars are added sequentially by the bifunctional enzyme WaaA (KdtA) before enzymes of the late Raetz pathway catalyze the transfer of two acyl chains to yield hexa-acylated Kdo_2_-lipid A. Core oligosaccharide sugars and phosphates are then sequentially added by their associated glycosyltransferases and kinases, respectively, to yield LPS. The ABC transporter MsbA flips the LPS molecule into the periplasmic-facing leaflet of the inner membrane where it is translocated by the Lpt system to the outer leaflet of the outer membrane (OM). Although absent in K-12 strains, O-antigen would be ligated to the terminal core oligosaccharide sugar in the periplasm prior to Lpt-dependent transport.

The mechanism of LPS/LOS synthesis (8, 9) has been studied quite extensively in *E. coli* K-12 and is modeled in Fig. 1. Synthesis of the lipid A component occurs on the cytoplasmic side of the inner membrane via the Raetz pathway. Like biogenesis of the peptidoglycan cell wall, initiation of the pathway requires an activated UDP-sugar, UDP-N-acetyl-glucosamine (UDP-GlcNAc). Early steps of the pathway result in the formation of a key precursor, lipid IV_A_, which is a *bis*-phosphorylated tetra-acylated disaccharide of glucosamine (GlcN). The enzyme WaaA, also known as KdtA, is then responsible for the transfer of 3-deoxy-D-*manno*-octulosonic acid (Kdo) residues to lipid IV_A_. These Kdo residues are actually part of the core oligosaccharide, but their presence is required for complete acylation of LPS as acyltransferases in the latter steps of the pathway require Kdo to generate the Kdo_2_-lipid A molecule (Fig. 1). This process is followed by the synthesis of the remaining core oligosaccharide, which in *E. coli* employs a variety of glycosyltransferases and kinases to sequentially add individual sugar and phosphate residues, respectively. Once the full-length lipid A-core molecule is generated, it is flipped across the inner membrane by the dedicated ABC transporter MsbA. Although absent in K-12 strains, O-antigen is added on the periplasmic side of the inner membrane. Mature LPS/LOS is then translocated by the lipopolysaccharide transport (Lpt) system to its final destination in the outer leaflet of the outer membrane (Fig. 1).

LPS/LOS is broadly considered essential in nearly all Gram-negative bacteria, but four organisms—*Neisseria meningitidis*, *Moraxella catarrhalis*, *Acinetobacter baumannii*, and most recently *Caulobacter crescentus*—have been demonstrated to survive in its absence (10–14). Among these organisms, however, *A. baumannii* is the only species shown to abandon the synthesis of its LOS as a resistance mechanism to LPS/LOS-targeting polymyxin antibiotics (13, 14). In conjunction with its non-fastidious growth and relevance as a highly antibiotic-resistant nosocomial pathogen, *A. baumannii* serves as a uniquely relevant tool to investigate mechanisms of outer membrane biogenesis and maintenance (15).

**Fig 2:**
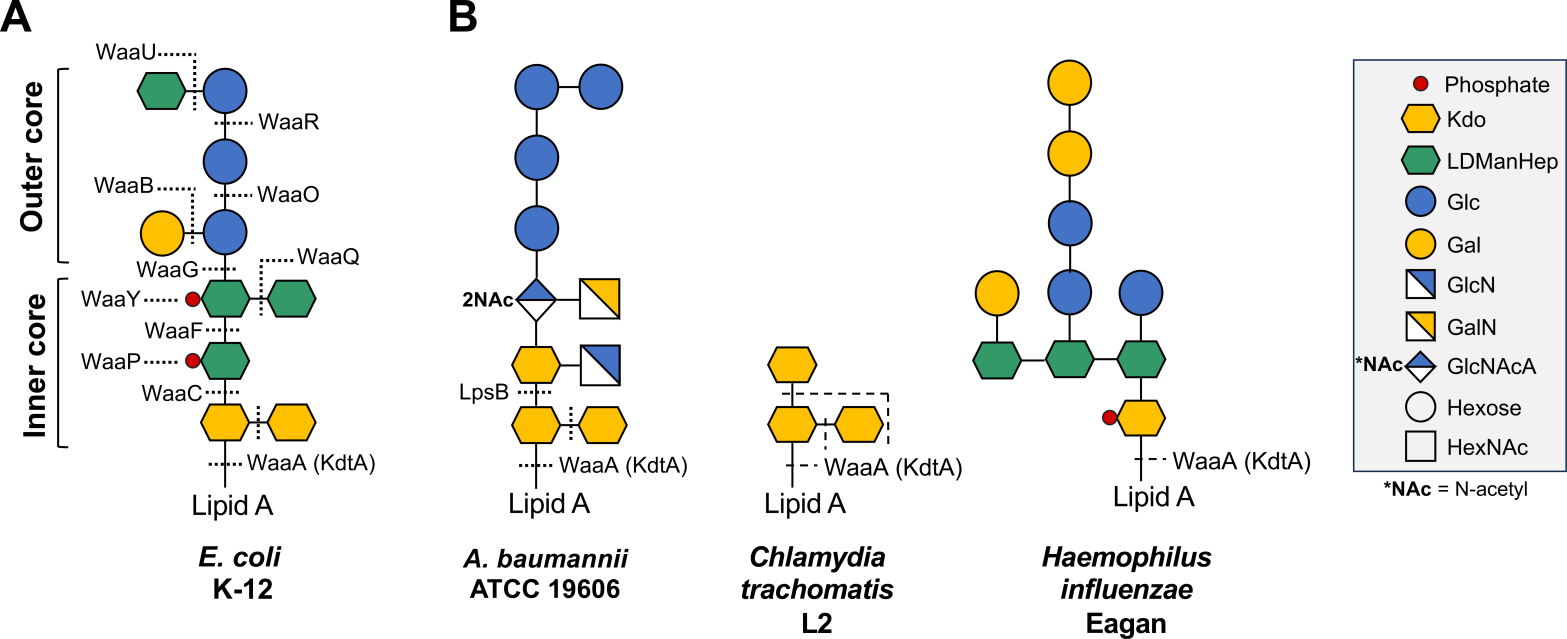
Composition of the LPS/LOS core oligosaccharide domains of select Gram-negative bacteria. (**A**) Structure of the core domain of *E. coli* K-12. The inner core region consists of two Kdo residues and several L,D-*manno*-heptoses. The outer core region contains three glucose residues, one galactose, and one L,D-*manno*-heptose. Dashed lines indicate the enzymes proposed to transfer each sugar or phosphate moiety. (**B**) Example of WaaA functionality in the assembly of different core oligosaccharides. Although bifunctional in *E. coli*, WaaA is trifunctional in *C. trachomatis* and monofunctional in *H. influenzae*. Core oligosaccharide residues are depicted using the official Symbol Nomenclature for Glycans.

Much progress has been made toward understanding the lipid A component of LOS in *A. baumannii* (9, 16, 17). However, experimental data examining the synthesis and impact of the remaining half of the LOS molecule—the core oligosaccharide—are limited. The core domain of most organisms, such as *E. coli* K-12, can be divided into outer core and inner core regions (Fig. 2A) (18). In *A. baumannii*, for which few structures have been solved to varying degrees, the outer core is typically quite variable among strains while the inner core is relatively conserved (19, 20). In all characterized organisms, WaaA is known to be solely responsible for sequential transfer of each successive Kdo residue in the LPS/LOS inner core. For example, WaaA of *E. coli* is bifunctional and will transfer two Kdo residues to the lipid A moiety sequentially (21). In organisms with a Kdo trisaccharide, such as *Chlamydia trachomatis*, WaaA is trifunctional with three sugars transferred to the lipid acceptor (Fig. 2B) (22). There are also examples of monofunctional Kdo transferases, like the WaaA of *Haemophilus influenzae* (23). Intriguingly, this does not appear to be the case in *A. baumannii*, as data show that disruption of the predicted glycosyltransferase *lpsB* results in a partially truncated core of two Kdo residues (24). This surprising truncation of the Kdo trisaccharide suggests a bifunctional *A. baumannii* WaaA homolog rather than the expected trifunctional enzyme—a departure from the convention set by all other characterized Gram-negative organisms. This phenomenon suggests that *A. baumannii* utilizes a novel mechanism for Kdo residue transfer.

In this report, we sought to better characterize the biosynthesis of the *A. baumannii* core oligosaccharide by examining the impact of predicted core oligosaccharide gene deletions on the *A. baumannii* ATCC 17978 core structure. In doing so, we have provided a toolset for targeted alteration of the core oligosaccharide structure that can be applied to a variety of commonly used lab strains. Our analysis also revealed an intriguing overlap of two gene deletions resulting in identical partial Kdo trisaccharide truncations, Δ*lpsB* and Δ*2903*, which we demonstrate have different effects on the integrity of the *A. baumannii* cell envelope. This chemotypic commonality suggests that WaaA is unexpectedly not responsible for the transfer of entire Kdo trisaccharide, providing the basis for a novel mechanism of Kdo transfer and core oligosaccharide synthesis in *A. baumannii*. We suggest here that this novel mechanism involves the use of both LpsB and 2903 to couple Kdo addition with the transfer of another seemingly unrelated core oligosaccharide sugar residue N-acetylglucosaminuronic acid (GlcNAcA).

## RESULTS

### Confirmation of predicted core locus of *A. baumannii* ATCC 17978

**Fig 3:**
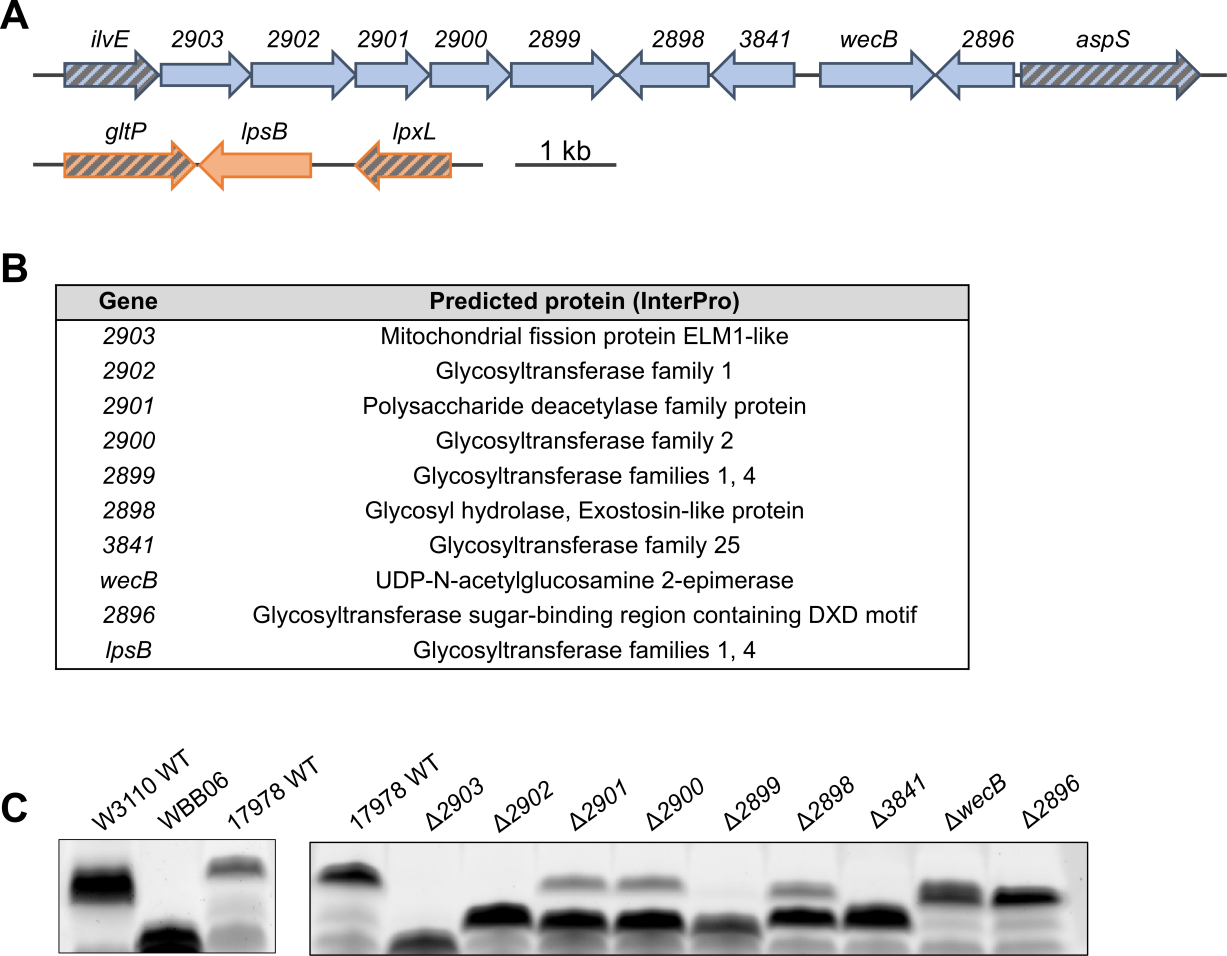
Confirmation of predicted core locus of *A. baumannii* 17978. (**A**) Organization of genes predicted to function in core oligosaccharide synthesis (26). Arrows indicate the directionality of transcription. Most predicted genes are present in a single locus between *ilvE* and *aspS* (top). *2903* through *2899* are predicted to be transcribed in a single unit. This locus also contains predicted hydrolases (*2901*, *2898*) and a predicted epimerase (*wecB*). At least one additional predicted glycosyltransferase, *lpsB*, has been implicated in core oligosaccharide biosynthesis (bottom). (**B**) Predicted proteins of genes implicated in core oligosaccharide biosynthesis generated via InterPro. (**C**) Analysis of core oligosaccharides via SDS-PAGE separation and ProQ Emerald 300 staining of proteinase K-treated cell lysates of indicated strains.

A cluster of genes predicted to participate in core oligosaccharide synthesis in *A. baumannii* was previously identified via *in silico* analysis by Kenyon and Hall (25). These suspected core oligosaccharide synthesis genes are consistently located between genes encoding the branched-chain amino acid aminotransferase *ilvE* and the aspartate-tRNA ligase *aspS*. While the contents of this locus differ among *A. baumannii* strains, the five-gene operon immediately downstream of *ilvE* is conserved among all strains with both published genomes and solved core oligosaccharide structures. To this effect, we focused our efforts on the common and amenable lab strain ATCC 17978, which possesses the core oligosaccharide type 2 locus (Fig. 3A).

As predicted by InterPro (27), this cluster contains an array of genes predicted to encode glycosyltransferases (*2902*, *2900*, *2899*, *3841*, *2896*) from various families as well as genes encoding a predicted polysaccharide deacetylase (*2901*), a glycosyl hydrolase (*2898*), a UDP-N-acetyl-glucosamine 2-epimerase (*wecB*), and an elongated mitochondrial1 (ELM1)-like fission protein (*2903*) (Fig. 3B). ELM1 family proteins are known to assist in ferrying dynamin-related proteins from the cytosol to mitochondrial fission sites in plants, but their role in bacteria remains unknown (28). A BLASTp search was relatively ineffective in revealing any known proteins related to 2903, so we instead utilized the 3D protein structure comparison server DALI in order to probe for any structural similarities between the predicted structure of 2903 and other known proteins (29). This overwhelmingly returned glycosyltransferase hits when the results were filtered to *E. coli* proteins of known structure including, but not limited to, the peptidoglycan GlcNAc transferase MurG, the colanic acid pyruvyltransferase WcaK, and the Kdo transferase WaaA (Table S1). This suggested that 2903 may have glycosyltransferase capabilities despite low amino acid similarity. Further supporting these proteins’ roles in core oligosaccharide synthesis, we found that localization predictions using the PSORTb bacterial protein subcellular localization prediction program placed most of these proteins in the cytoplasm (30) (Table S2). PSORTb was unable to confidently assign a subcellular location for 2903 and 2901; however, the lack of any secretory signals or transmembrane domains in the amino acid sequences suggests that they are likely to function at the cytoplasmic face of the inner membrane.

Clean deletions were generated for each individual gene in the predicted core locus, and the effect on core oligosaccharide structure was evaluated. Proteinase K-treated whole-cell lysates of each mutant and the 17978 wild-type parent were separated by SDS-PAGE using a 4%–12% Bis-Tris gel and the LOS pattern was visualized by staining with Pro-Q Emerald 300 carbohydrate dye (Fig. 3C). The *E. coli* strain W3110 was used as a control to exemplify full-length wild-type core oligosaccharide, while *E. coli* strain WBB06 represented the most severely truncated chemotype option containing only two Kdo residues. Using these two chemotype extremes as controls, it was apparent that the *A. baumannii* 17978 wild type displayed a heterogenous banding pattern divided into full-length core oligosaccharide (top band) and truncated intermediate chemotypes (other bands) that are typically seen in *A. baumannii* (31, 32). As predicted, each mutant exhibited a variation of this standard banding pattern indicating a truncation of the core oligosaccharide, thereby implicating each gene in core oligosaccharide synthesis.

We also determined if truncation of the core oligosaccharide altered the structure of the lipid A anchor of LOS for each mutant. To do this, strains were radiolabeled using ^32^P_i_ and the ^32^P-lipid A species evaluated by thin-layer chromatography (TLC) as previously described [Fig. S1, (16)]. As expected, wild-type 17978 produced four major lipid A species. *A. baumannii* synthesizes both hexa- and hepta-acylated lipid A with the hepta-acylated form as the dominant species (16). Each acylated form can be modified further with an additional hydroxyl group yielding a total of four major lipid A species. No matter the core truncation, we found that all mutants produced the same four lipid A species similar to wild type.

### Verification of core oligosaccharide mutants

**Fig 4:**
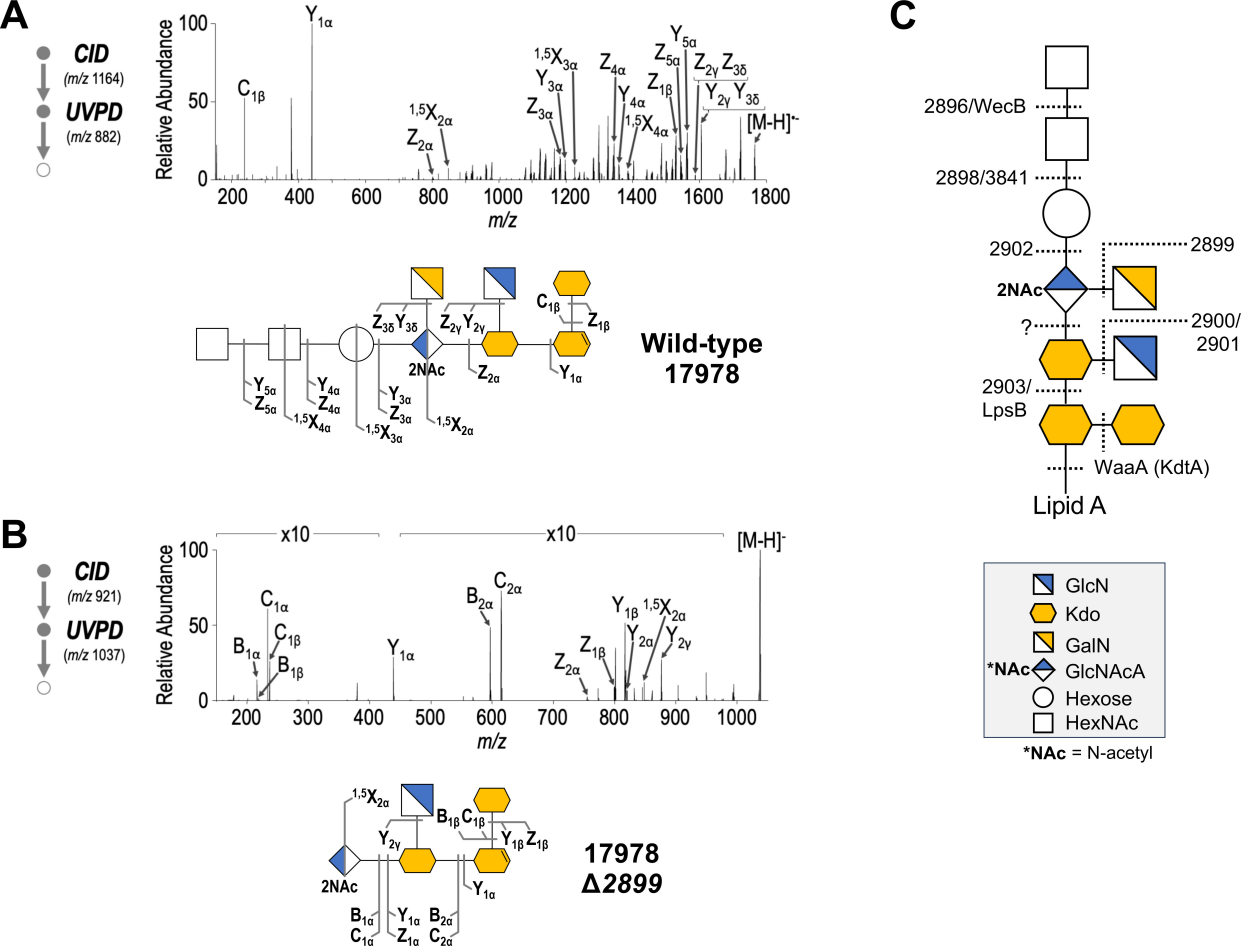
Structural characterization of *A. baumannii* core oligosaccharides by UVPD mass spectrometry. For each purified LOS, low-energy collision-induced dissociation was used to cleave the lipid A and oligosaccharide substructures of LOS from each other. This was followed by a high-resolution ultraviolet photodissociation method to provide MS^3^ data. (**A**) 17978 wild-type core oligosaccharide. CID of the precursor ion of *m/z* 1,164.57 (*z* = 3) followed by UVPD of the fragment ion of *m/z* 882.79 (*z* = 2). (**B**) Representative knockout mutant 17978 Δ*2899* core oligosaccharide. CID of the precursor ion of *m/z* 921.47 (*z* = 3) followed by UVPD of the fragment ion of *m/z* 1,037.29 (*z* = 1). H_2_O loss from Kdo upon cleavage of the glycosidic bond between the core oligosaccharide and lipid A during CID is depicted as a double bond on the Kdo symbol. (**C**) Major chemotypes of respective knockout mutants of each gene in the predicted core locus. All chemotypes and corresponding MS^3^ analysis can be found in Fig. S2a through j.

**Fig 5:**
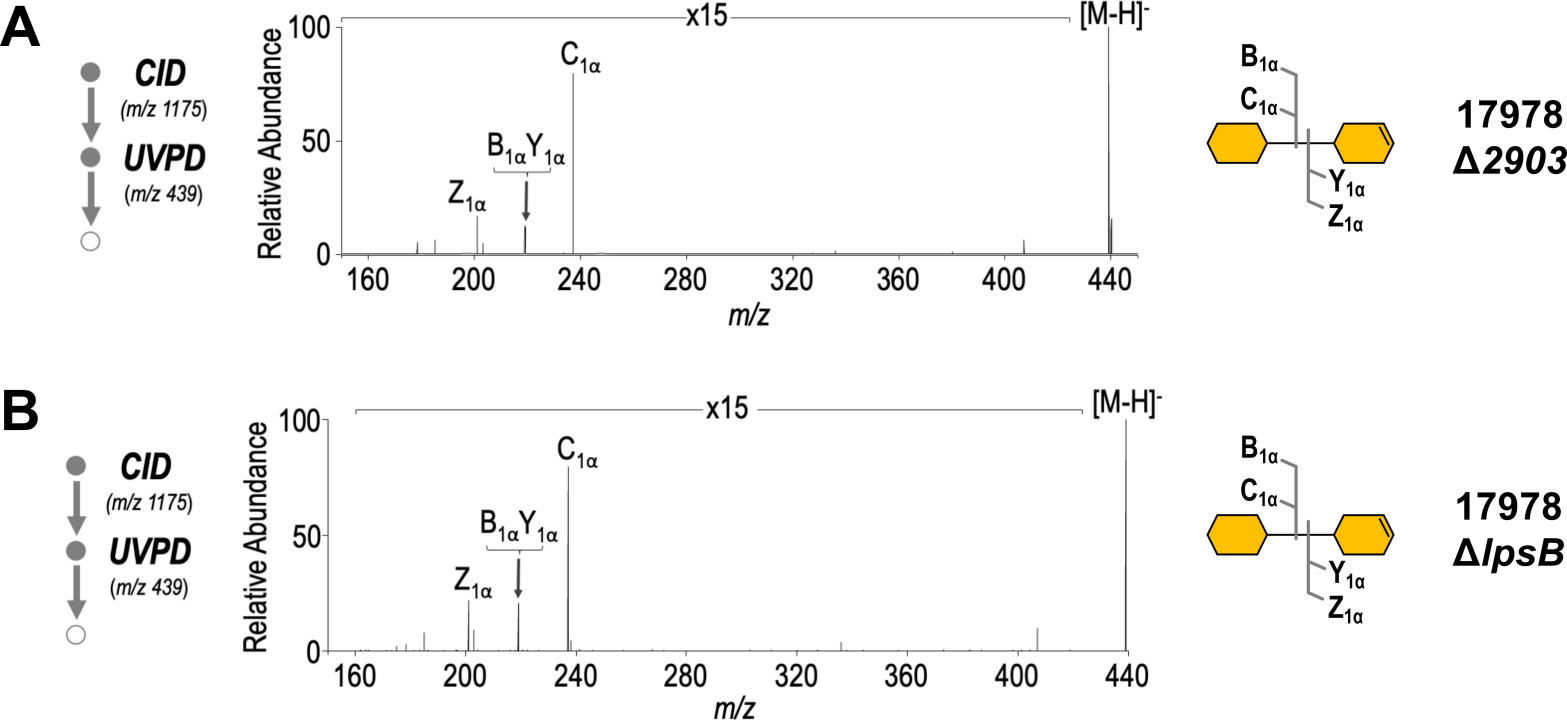
Δ*2903* and Δ*lpsB* share identical core chemotypes. (**A**) MS^3^ analysis of core oligosaccharide from *A. baumannii* Δ*2903*, which has a core consisting of two Kdo residues. CID of the precursor ion of *m/z* 1,174.7041 (*z* = 2) followed by UVPD of the fragment ion of *m/z* 439.1105 (*z* = 1). The corresponding MS^3^ analysis can be found in Fig. S2b. (**B**) MS^3^ analysis of core oligosaccharide from *A. baumannii* Δ*IpsB*, which has a core consisting of two Kdo residues. CID of the precursor ion of *m/z* 1,174.6995 (*z* = 2) followed by UVPD of the fragment ion of *m/z* 439.1091 (*z* = 1). The corresponding MS^3^ analysis can be found in Fig. S3.

While SDS-PAGE analysis serves as a useful way to observe basic changes in core oligosaccharide structure, it does not provide the necessary resolution to define changes in residue number or identity. In pursuit of a more comprehensive characterization of these chemotypes, we extracted LOS from each strain and the wild type via a modified hot phenol-water method to yield purified, intact LOS molecules (20). We then subjected the purified LOS to multi-stage tandem mass spectrometry (MS^3^) in which the first stage is the conventional collision-induced dissociation (CID), a methodology that disassembles the lipid A component from the core oligosaccharide and generates a few other diagnostic fragment ions. This is followed by ultraviolet photodissociation (UVPD) of core oligosaccharide species to generate a plethora of fragment ions and detailed structural information for each LOS chemotype. With this strategy, CID cleaves the core oligosaccharide from the lipid A portion and allows comprehensive characterization of the oligosaccharide structure by UVPD without creating confounding or overlapping fragment ions from the lipid A portion. Each core oligosaccharide mutant was analyzed using this method and the associated chemotypes were illustrated using the official Symbol Nomenclature for Glycans (SNFG) (33) (Fig. 4 and 5; Fig. S2).

The wild-type LOS was first analyzed via CID of the *m/z* 1,164.57 (z = 3) precursor ion corresponding to intact LOS followed by UVPD of the resulting *m/z* 882.79 (z = 2) fragment ion corresponding to the core oligosaccharide (Fig. 4A). As shown in the SNFG illustration, the full-length wild-type chemotype is composed of nine sugar residues. This structure was identical to that of the previously characterized strain AB5075, which was expected as this strain shares a homologous core oligosaccharide locus with ATCC 17978 (20). Importantly, while the MS^3^ method used in this study can offer a detailed look at core oligosaccharide structure with minimal sample preparation, it is unable to distinguish between stereoisomers. The first six residues of the core oligosaccharide described here are specifically assigned based on comparisons to previously published NMR analysis of the core oligosaccharide of *A. baumannii* strain ATCC 19606, which shares an identical inner core oligosaccharide structure as well as homologs of the genes apparently involved in inner core oligosaccharide synthesis (19, 25). Despite a sizeable region of homology, strains 17978 and 19606 possess different core loci (26). The outermost three residues of strain 17978, therefore, differ from the 19606 structure and are only identifiable as hexose (Hex) and N-acetylhexosamine (HexNAc) residues due to MS^3^ limitations.

In comparison to the full-length wild type, LOS from Δ*2899*—the chromatogram for which is shown as a representative to demonstrate the technique in a core truncation mutant—possesses an intermediate chemotype of only five sugar residues, lacking the outermost three residues and the branching galactosamine (GalN) (Fig. 4B). This detailed analysis was repeated for each mutant in the core oligosaccharide locus (Fig. S2a through j). The unique core truncations resulting from each directed mutation along with predicted protein function allowed us to begin assigning putative functions to each gene product. The simplest proteins for which we can assign functions are the predicted glycosyltransferases that do not work in conjunction with any other core oligosaccharide locus members. That is, 2899 is likely responsible for GalN addition, while 2902 is responsible for outer core Hex transfer as deletion of either gene results in the lack of the corresponding residue. 3841 is presumably responsible for the transfer of the first HexNAc in the outer core. Our analysis implicates the predicted hydrolase 2898 in addition of this sugar residue as well, but its particular role is unclear. As deletion of either the predicted deacetylase 2901 or the predicted glycosyltransferase 2900 both result in the lack of a branching glucosamine (GlcN) residue, it is likely that 2901 and 2900 work together to generate and transfer the GlcN residue. Similar logic allows us to deduce that the substrate donor for the terminal HexNAc residue would be generated by the predicted UDP-GlcNAc 2-epimerase WecB and transferred by the predicted glycosyltransferase 2896. Despite our identification of putative glycosyltransferases for all other core sugar residues, a gene responsible for N-acetylglucosaminuronic acid transfer was not immediately obvious.

### Δ*2903* and Δ*lpsB* share identical core oligosaccharide chemotypes but remain phenotypically distinct

As previously discussed, organisms with more than one Kdo in succession with the inner core exclusively rely on the Kdo transferase WaaA (KdtA) to transfer all inner core Kdo residues. However, deletion of *lpsB*, which encodes a predicted glycosyltransferase (Fig. 3A), was previously reported to yield a Kdo_2_ core chemotype—a partial truncation of the typical Kdo trisaccharide—suggesting that WaaA is not responsible for the transfer of all three Kdo residues in *A. baumannii* (24). We were therefore surprised to find yet another mutant in our CID/UVPD MS^3^ analysis, Δ*2903*, with a core domain of only two Kdo sugars (Fig. 5A; Fig. S2b). Although the lack of information on the roles of ELM1-like mitochondrial fission proteins in bacteria makes it difficult to confidently assign its function in core oligosaccharide synthesis, the structural similarity of the 2903 protein to known glycosyltransferases, including WaaA, suggests that it may also be involved in the transfer of the third Kdo sugar. To first confirm that both mutants possessed the same chemotypes, we conducted CID/UVPD MS^3^ analysis on intact purified LOS from 17978 Δ*lpsB* (Fig. 5B; Fig. S3) and compared the core oligosaccharide structure to that of Δ*2903*. Both mutants yielded nearly indistinguishable spectra, making it clear that the *2903* and *lpsB* mutants have identical chemotypes (Fig. 5). This finding was also confirmed in a second genetic background with deletion of genes encoding homologs of *lpsB* or *2903* in *A. baumannii* strain 19606 resulting in identical LOS chemotypes via SDS-PAGE (Fig. S4).

**Fig 6:**
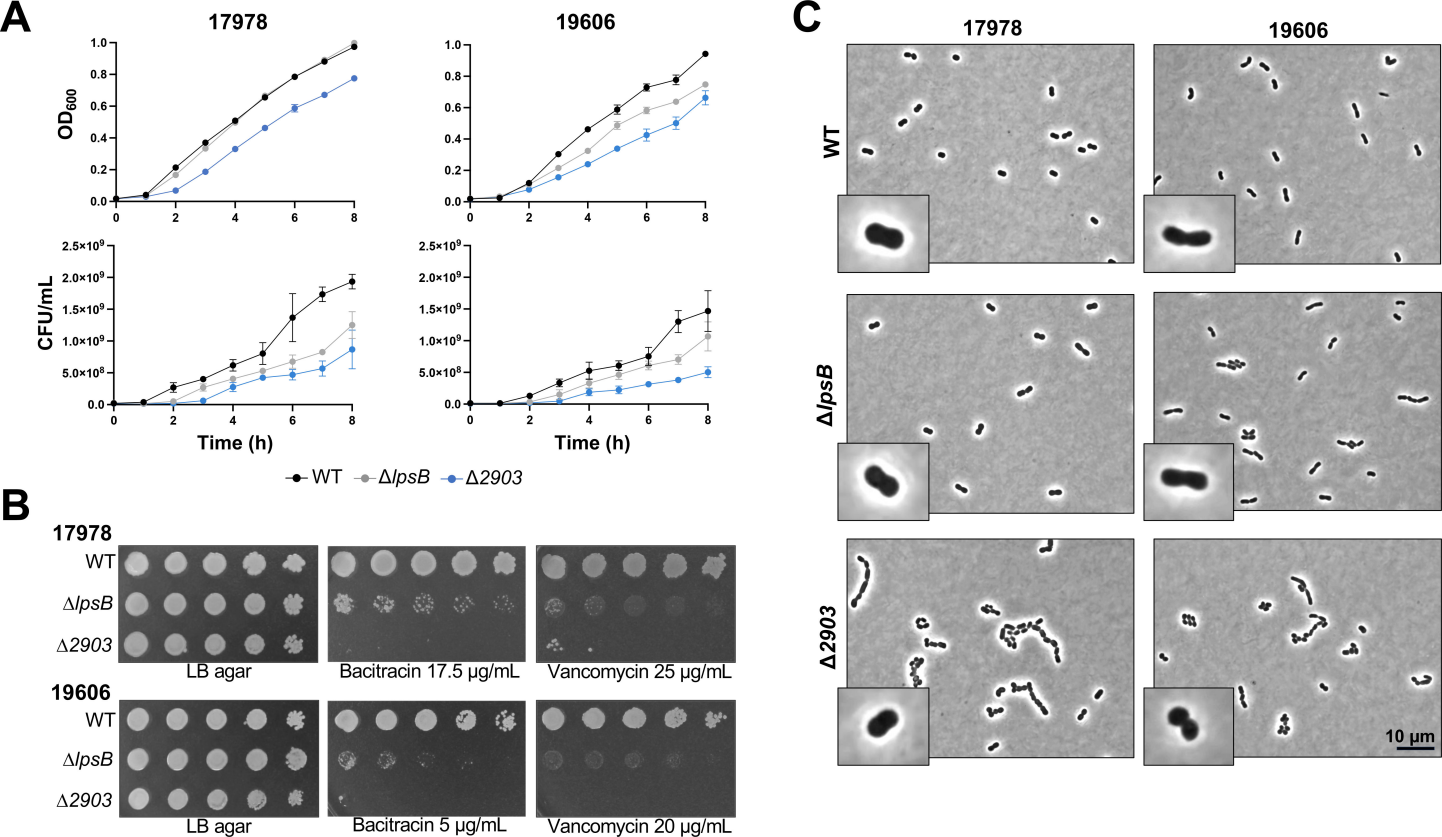
Δ*2903* and Δ*lpsB* phenotypes suggest varying degrees of cell wall synthesis defects. (**A**) Growth of wild-type 17978 or 19606 with their corresponding Kdo_2_ oligosaccharide chemotype mutants at 37°C was evaluated by both OD_600_ and serial dilutions to calculate CFU/mL. Error bars represent standard deviation with technical triplicates. Data are representative of three biological replicates. (**B**) Comparison of antibiotic sensitivity between *A. baumannii lpsB* and *2903* mutants by efficiency of plating assays. Serial dilutions of the indicated strains were spotted to LB agar plates containing 17.5 µg/mL bacitracin and 25 µg/mL vancomycin (17978 strains) or 5 µg/mL bacitracin and 20 µg/mL vancomycin (19606 strains). (**C**) Cell morphology of mid-log phase cultures using phase contrast microscopy (100× magnification). Insets are 3.75× zoom of larger images.

It is reasonable to assume that these identical chemotypes would yield similar effects on overall cell envelope integrity in both Δ*lpsB* and Δ*2903*. Instead, we realized that these two mutants are phenotypically distinct. Comparisons of growth patterns by OD_600_ in broth cultures among the mutants and parents made it clear that 17978 Δ*2903* had a growth defect not seen in Δ*lpsB* (Fig. 6A). This defect was even more severe when identical mutants were tested in a 19606 background. When growth was measured by colony-forming units (CFU), however, a growth defect was evident for both 17978 and 19606 *lpsB* mutants. Still, this ∆*lpsB* defect was not as severe as the growth defect of *2903* mutants.

We then turned to the use of the cell wall-targeting antibiotics vancomycin and bacitracin to probe cell envelope integrity via efficiency of plating assays. These large peptide antibiotics are commonly used to measure outer membrane permeability as their chemical properties typically prevent them from passing through an intact outer membrane (31). Although both 17978 ∆*lpsB* and ∆*2903* were less resistant to both antibiotics compared to wild type as demonstrated by their diminished growth, the *2903* mutant was far more sensitive compared to its Δ*lpsB* counterpart (Fig. 6B). This clear increase in antibiotic sensitivity was also observed for *lpsB* and *2903* mutants in the 19606 background. Note that lower concentrations of bacitracin and vancomycin were used for 19606 strains, as wild-type 19606 shows a slightly higher sensitivity to these antibiotics compared to 17978.

To investigate this incongruity further, we evaluated morphological differences by phase contrast microscopy during mid-log phase (Fig. 6C). The 17978 wild-type and Δ*lpsB* cultures could be visualized as archetypal coccobacilli. However, the *2903* mutant on average appeared slightly shorter in length. Δ*2903* cells also often formed chains and clusters that were not seen in the parent and very rarely observed in the 17978 *lpsB* mutant. These Δ*2903* phenotypes were even more pronounced when microscopy was conducted in 19606 due to its characteristically longer cell shape compared to 17978. Although still not as severe as in Δ*2903*, the chaining observed in a *lpsB* mutant was also more prominent in this genetic background. This was unsurprising as this mutant also displayed slowed growth. Cell measurements showed a significant change in cell length upon deletion of *2903* (*P*-value ≤ 0.0001) in both genetic backgrounds (Fig. S5). Only in 19606 were changes in cell width observed and were found to be significant for both Δ*2903* and Δ*lpsB*. Altogether, the morphological changes observed in the *2903* mutants, and to a lesser degree *lpsB* mutants, indicate defects in cell division as the cells are clearly unable to grow and divide properly (34, 35).

### Co-expression of *2903* and *lpsB* results in addition of KdoIII and GlcNAcA to core oligosaccharide

Our analysis thus far suggested that strains lacking *2903* or *lpsB*—two mutants proposed to be involved in KdoIII residue addition to the core oligosaccharide—exhibited defects in cell division to varying extents. Cell division errors much like those observed here are often found in cells with underlying defects in peptidoglycan cell wall synthesis (35–37). It was unclear why this might be the case as there is no known overlap between core oligosaccharide synthesis and cell wall synthesis. However, a potential explanation became more apparent when we recalled that a GlcNAcA transferase candidate was not identified in our initial analysis of core oligosaccharide mutant chemotypes (Fig. 4C). The UDP-GlcNAcA substrate is predicted to be generated via dehydrogenase activity at the C-6 position of UDP-GlcNAc by Gna, a homolog of WbpA in *Pseudomonas aeruginosa* and TviB in *Salmonella enterica* serovar Typhi (25, 38, 39). Previous reports have shown that the deletion of *gna* along with a downstream UDP-GlcNAcA C-4 epimerase gene, *gne2*, results in partial truncation of the core oligosaccharide (40). Importantly, this GlcNAc precursor is an essential component of the peptidoglycan cell wall (41). It is possible that the disruption of LOS synthesis via deletions of *2903* or *lpsB* results in an imbalance of this shared precursor, leading to a defect in peptidoglycan synthesis that is apparently more severe in ∆*2903* based on its exaggerated phenotypic defects compared to ∆*lpsB*. This shared GlcNAc precursor, in conjunction with the curious lack of an identified *A. baumannii* GlcNAcA transferase and the cell division defects exhibited by mutants with a Kdo_2_-lipid A chemotype, led us to hypothesize whether it was possible that 2903 and LpsB were required for the transfer of both KdoIII and GlcNAcA residues.

**Fig 7:**
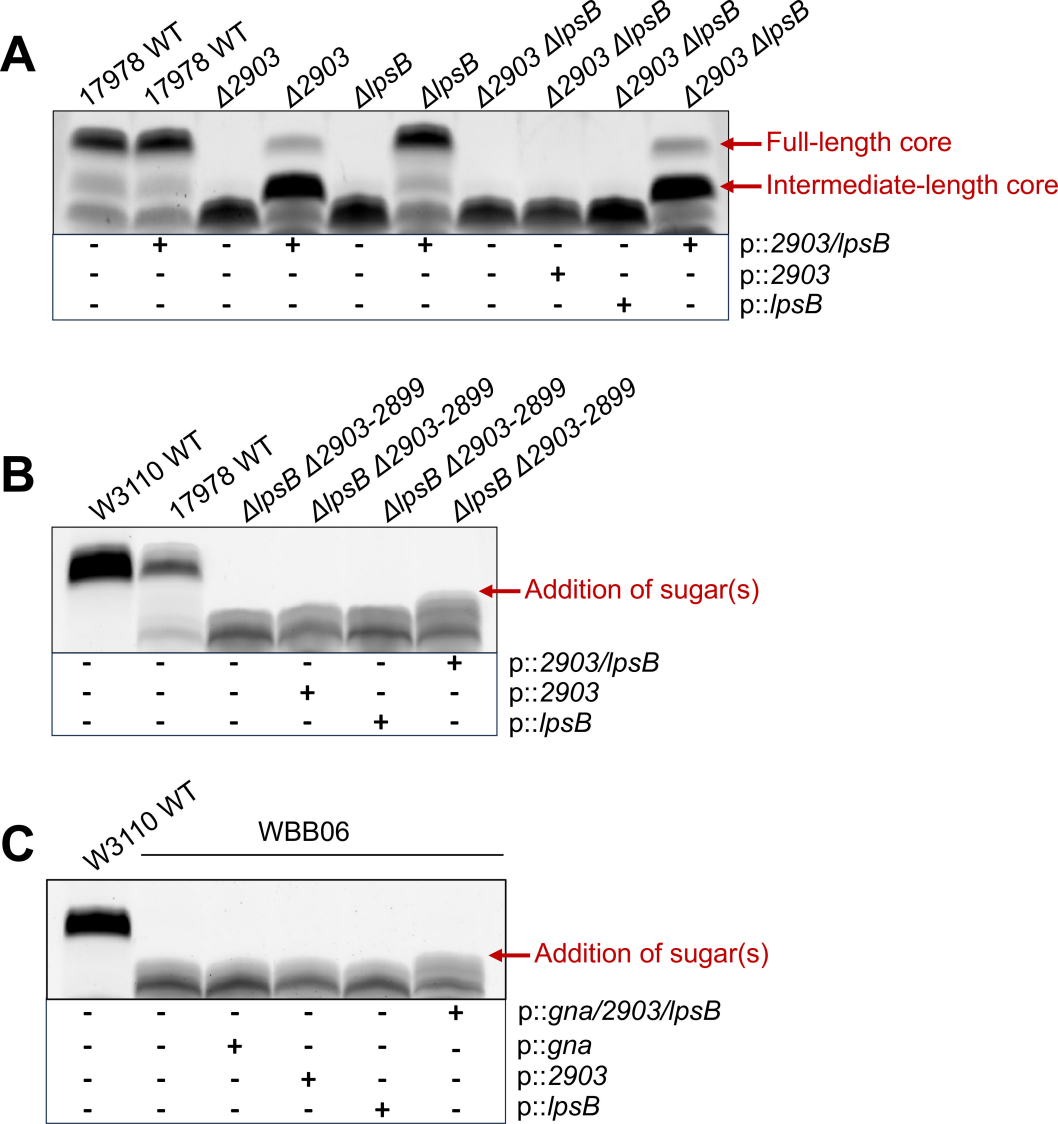
Co-expression of 2903 and LpsB results in alteration of *A. baumannii* LOS and *E. coli* LPS core oligosaccharide. Indicated strains were induced with 25 µM IPTG overnight in 5 mL LB broth. Core oligosaccharide profiles were analyzed via SDS-PAGE separation with 4%–12% Bis-Tris (**A**) or 16% Tricine (**B and C**) of proteinase K-treated whole-cell lysates followed by ProQ Emerald 300 staining. (**A**) Effect of 2903 and LpsB co-expression in an *lpsB*, *2903* double mutant. (**B**) Effect of 2903 and LpsB co-expression in *A. baumannii* Δ*lpsB*Δ*2903–2899*, a mutant lacking all known confounding core oligosaccharide transferases. (**C**) Heterologous co-expression of both 2903 and LpsB, along with the enzyme Gna that is required for UDP-GlcNAcA synthesis, results in sugar addition to the inner core oligosaccharide of *E. coli* WBB06.

To determine if this was the case, we co-expressed *2903* and *lpsB* using the isopropyl β-D-1-thiogalactopyranoside (IPTG)-inducible vector pMMB. As shown by SDS-PAGE analysis, the expression of both proteins resulted in restoration of full-length core oligosaccharide in *2903* and *lpsB* single mutants (Fig. 7A). Our co-expression plasmid was also able to restore synthesis of a full-length core species in the *2903 lpsB* double mutant successfully (top band). We did find, however, that complementation of strains lacking a chromosomal copy of *2903* was only partial with the production of both wild-type LOS and accumulation of LOS with a core oligosaccharide of intermediate length (intermediate band), possibly due to a stoichiometric imbalance of *2903* and *lpsB* or later core oligosaccharide synthesis enzymes.

**Fig 8:**
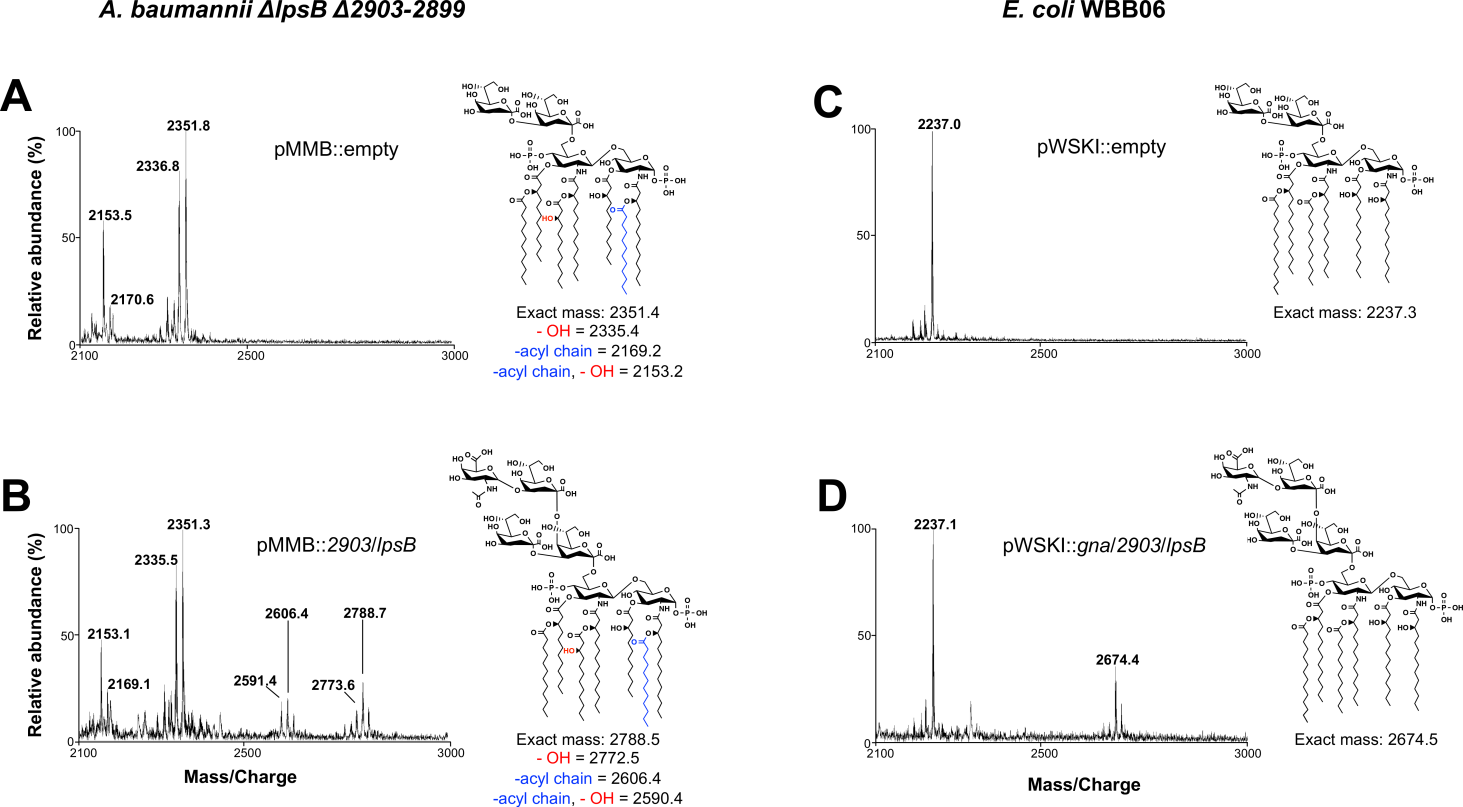
Negative-ion MALDI-TOF mass spectrometry analysis confirms addition of KdoIII and GlcNAcA by LpsB and 2903. LOS/LPS samples of the indicated strains were isolated via acidified Bligh-Dyer followed by anion-exchange chromatography to remove contaminating glycerophospholipids. Samples were then analyzed by MALDI-TOF mass spectrometry using ATT matrix. Representative LOS/LPS structures are displayed next to the corresponding spectrum. For *A. baumannii*, an additional hydroxyl group (red) can be found on the 2′-linked secondary acyl chain. Also, a portion of *A. baumannii* LOS contains an additional acyl chain (shown in blue) giving a hepta-acylated lipid A. (**A**) *A. baumannii* ∆*lpsB*∆*2903–2899* pMMB::empty produced two major ions at *m/z* 2,351.8 and 2,336.8 corresponding to hepta-acylated Kdo_2_-lipid A species with and without the additional -OH group, respectively. Peaks found at *m/z* 2,170.6 and 2,153.5 correspond to hexa-acylated species with and without the hydroxylated fatty acid, respectively. (**B**) *A. baumannii* ∆*lpsB*∆*2903–2899* pMMB::*2903*/*lpsB* produced similar Kdo_2_-lipid A species compared to panel **A** as well as additional species found at *m/z* 2,788.7 and 2,773.6 representing GlcNAcA-Kdo_3_-hepta-acylated lipid A species. Peaks at *m/z* 2,606.4 and 2,591.4 represent hexa-acylated variants of this structure. (**C**) *E. coli* WBB06 pWSKI::empty produced one major ion at *m/z* 2,237.0 representing hexa-acylated Kdo_2_-lipid A. (**D**) *E. coli* WBB06 pWSKI::*gna/2903/lpsB* produced a similar major ion as found in panel **C** but contained an additional species at *m/z* 2,674.4 representing GlcNAcA-Kdo_3_-hexa-acylated lipid A.

Once we established functionality of our *2903*/*lpsB* co-expression vector, we sought to conduct a selective core oligosaccharide reconstitution analysis to observe LOS structural changes when only *2903* and *lpsB* were present for inner core assembly. A mutant lacking gene *2903–2899* in the major core locus and *lpsB* was generated yielding a Kdo_2_ core chemotype. We again conducted SDS-PAGE analysis using a 16% Tricine gel, which provides better resolution of minor structural changes (e.g., addition of a single sugar). In this analysis, no changes in band migration were seen when only *2903* or *lpsB* was expressed in the mutant alone, but a very minor upward shift in band migration (red arrow, Fig. 7B) was evident when the two genes were co-expressed. This suggested that only one to two sugars were being added to the core oligosaccharide. To confirm this suspected chemotype, core-lipid A from Δ*lpsB*Δ*2903–2899* co-expressing *2903* and *lpsB* was isolated via an acidified Bligh-Dyer extraction and purified from contaminating glycerophospholipids via anion-exchange chromatography. The purified molecule was assessed by matrix-assisted laser desorption/ionization-time of flight (MALDI-TOF) mass spectrometry (Fig. 8).

The spectrum generated by Δ*lpsB*Δ*2903–2899* with empty vector revealed the presence of a major ion at *m/z* 2,351.8, which corresponds to the expected mass of *bis*-phosphorylated, hepta-acylated Kdo_2_-lipid A containing 2-hydroxylauric acid (Fig. 8A). The remaining major peaks correspond to hepta-acylated Kdo_2_-lipid A lacking the additional hydroxyl group (*m/z* 2,336.8) and hexa-acylated Kdo_2_-lipid A variants (*m/z* 2,153.5, *m/z* 2,170.6). These results are consistent with previous studies that have indicated that *A. baumannii* produces a mixture of hexa- and hepta-acylated lipid A (16, 42). The spectrum collected from Δ*lpsB*Δ*2903–2899* co-expressing *2903* and *lpsB* also displayed similar peaks corresponding to hexa- and hepta-acylated lipid A with and without 2-hydroxylauric acid as expected (Fig. 8B). Remarkably, *2903* and *lpsB* co-expression also resulted in the appearance of additional species matching the exact masses of hexa- and hepta-acylated GlcNAcA-Kdo_3_-lipid A (*m/z* 2,606.4 and *m/z* 2,788.7). In agreement with our hypothesis, this confirmed the addition of both Kdo and GlcNAcA residues to the *A. baumannii* core oligosaccharide by 2903 and LpsB.

A similar approach was taken in *E. coli* strain WBB06 in which *2903* and *lpsB* were heterologously co-expressed from the IPTG-inducible vector pWSKI (43). In this strain, genes encoding the inner core heptosyltransferases WaaC and WaaF have been disrupted. As in the *A. baumannii* Δ*lpsB*Δ*2903–2899* mutant, WBB06 possesses a Kdo_2_ core oligosaccharide (44). Since *E. coli* lacks the innate ability to synthesize GlcNAcA, we expressed *gna* alongside *2903* and *lpsB* to facilitate the synthesis and transfer of a GlcNAcA residue to the core. No changes in band migration were seen when *gna*, *2903*, or *lpsB* was individually expressed according to SDS-PAGE (Fig. 7C). In comparison, expression of *gna, 2903,* and *lpsB* in concert resulted in an upward shift in band migration (red arrow), mimicking the minor core extension seen in the *A. baumannii* system. MALDI-TOF mass spectrometry of core-lipid A species isolated from WBB06 pWSKI showed a major ion corresponding to hexa-acylated Kdo_2_-lipid A (*m/z* 2,237.0) in accordance with prior literature as well as our SDS-PAGE results (Fig. 8C) (45). The spectrum generated from WBB06 expressing *gna*, *2903*, and *lpsB,* however, showed an additional species at *m/z* 2,674.4 corresponding to a GlcNAcA-Kdo_3_ core attached to *E. coli* lipid A (Fig. 8D). The reconstitution of this partial *A. baumannii* inner core in *E. coli* strongly supports that the KdoIII and GlcNAcA residues are transferred in unison and require both LpsB and 2903.

## DISCUSSION

**Fig 9:**
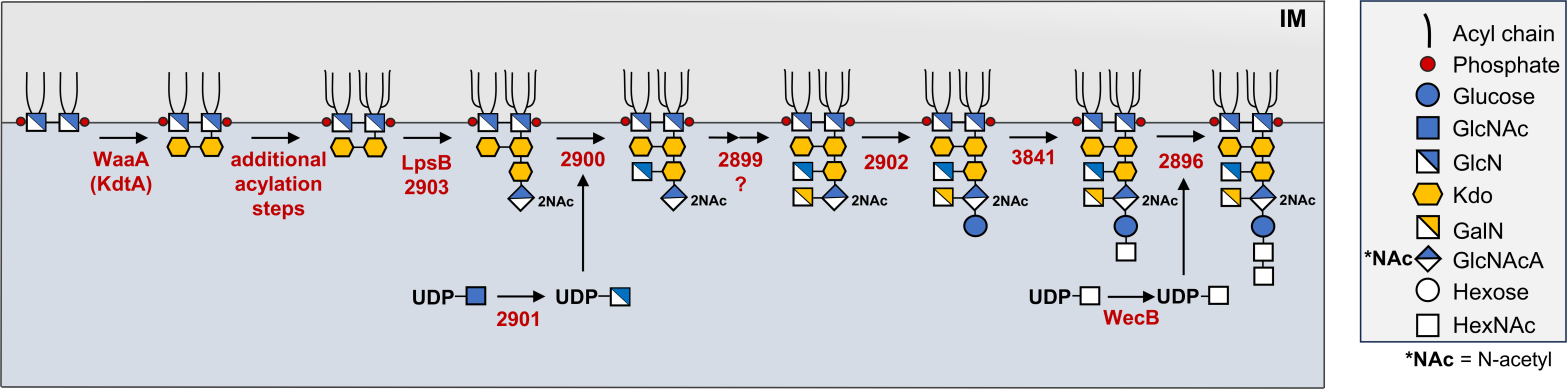
Model of core oligosaccharide synthesis in *A. baumannii*. All core oligosaccharide residue additions following WaaA activity and additional acylation steps are based upon putative protein functions as predicted by InterPro, detailed structure analysis of *A. baumannii* core synthesis mutants, and our *lpsB*/*2903* co-expression experiments in both *A. baumannii* and *E. coli*. Notably, a deacetylase not identified here is likely required for addition of the GalN residue. 2898, a predicted glycosyl hydrolase proven to play a role in *A. baumannii* outer core oligosaccharide synthesis, is not depicted as its function is unclear.

The analysis described here makes it evident that *A. baumannii* deviates from the conventional model of core oligosaccharide synthesis that has long been established in Gram-negative bacteria. Our assessment of the chemotypes of each core truncation mutant allows us to construct a model of this unique organism’s core oligosaccharide synthesis (Fig. 9). The proposed mechanism begins with the addition of only two Kdo residues by WaaA to the lipid A precursor, lipid IV_A_. This is followed by additional acylation steps to yield a mixture of hexa- and hepta-acylated lipid A molecules that is characteristic of *A. baumannii* LOS (16). Based on our findings, this is followed by the co-dependent transfer of both KdoIII and GlcNAcA residues in a process that, at minimum, requires both LpsB and 2903. Deletion of either 2900 or 2901 resulted in the complete loss of the GlcN residue, suggesting a straightforward mechanism of 2901-mediated deacetylation of UDP-GlcNAc to UDP-GlcN followed by transfer to the KdoIII residue by 2900. At this point, our structural work indicated that a *2899* mutant resulted in a loss of the GalN and outer core residues, which would normally implicate 2899 in simple transfer of GalN to the core. However, when put into context the complete structural data set, it is apparent that this step is likely more nuanced. Several unrelated core oligosaccharide mutants possessed GalNAc residues at this position in lieu of the expected GalN that is exclusively present in the full-length wild-type structure (Fig. S2a). Indeed, this finding is corroborated by a previous NMR study of strain 19606 in which a GalNAc residue was occasionally found to replace the GalN in truncated variations (19). If a UDP-GalNAc residue were to be deacetylated prior to addition to the core, this would necessitate substrate promiscuity by the 2899 transferase to explain the mixture of GalN and GalNAc in some core oligosaccharides. This cannot be the case, however, as only the GalN residue is present in the full-length wild-type core. It is therefore more likely that UDP-GalNAc is transferred to the growing core by 2899 and subsequently deacetylated by an unidentified enzyme, a mechanism that has been observed in other organisms (46, 47). This presumed deacetylation step would conclude inner core synthesis.

Precise assignments of the 17978 inner core residues are based on homology to 19606 as the two strains share homologs of all inner core-related genes (25). The same is true of the initial glucose residue of the outer core, proposed here to be transferred by 2902. Beyond this, however, the core loci of the two strains diverge and comparisons to the pre-existing NMR data can no longer be made. The CID/UVPD MS technique used for our analyses is unable to distinguish stereoisomers and thus leaves the exact identities of the remaining outer core residues unnamed. With this in mind, we propose that 3841 transfers the first outer core HexNAc. This is followed by 2896-driven transfer of a second HexNAc residue that requires the predicted epimerase WecB for formation of the UDP-activated sugar donor. This prediction is based on our finding that a *wecB* deletion results in either an absent or errant terminal sugar addition (Fig. S2i). Previous reports have indicated that the *wecB* gene at this locus encodes a protein with 57% identity to the GlcNAc 2-epimerase WecB of *Psychrobacter*, indicating that this protein also generates a ManNAc residue from the epimerization of GlcNAc (26). With more precise techniques, it is likely that this terminal sugar will be designated a ManNAc.

Although deletion of the predicted glycosyl hydrolase 2898 resulted in a partial truncation of the two outermost HexNAc residues, suggesting that it acts on the residue transferred by 3841, its actual role in core oligosaccharide synthesis remains unclear. Its annotation suggests that it would hydrolyze the glycosidic linkage of a different oligo- or polysaccharide to generate the substrate for the 3841 transferase. However, a physiological justification for this is doubtful, as it is unlikely that complete outer core oligosaccharide synthesis would be dependent upon breakdown of another cellular component. Further complicating the role of 2898 in core synthesis is a previous finding that this gene appears to be conserved across many *A. baumannii* strains, even in those lacking any HexNAc residues in the outer core region (19, 26). The uncertainty surrounding this cryptic protein and a clear need for a more dedicated investigation into its function have led us to omit 2898 from the core oligosaccharide synthesis model, but it certainly presents an interesting challenge for future studies. Nevertheless, our work lends itself to a comprehensive model of core oligosaccharide synthesis in *A. baumannii* that has not yet been defined and can be extrapolated to many *Acinetobacter* strains sharing even partial core oligosaccharide locus homology.

By far the most unique part of *A. baumannii* core assembly uncovered here is its apparently novel mechanism of KdoIII and GlcNAcA transfer by LpsB and 2903. Reconstitution of the core oligosaccharide showed that LpsB and 2903 are not able to function alone but instead rely on each other to catalyze the addition of both the KdoIII and GlcNAcA residues. Our data support a number of interpretations regarding the complete mechanism of this dual-residue transfer. The simplest model is one in which 2903 and LpsB are both glycosyltransferases that form a complex allowing for transfer of both residues in quick succession. It is also hypothetically possible that one protein forms a Kdo-GlcNAcA disaccharide precursor that is then transferred to the Kdo_2_-lipid A substrate by the other protein. Either model would explain the dependency on one another for KdoIII/GlcNAcA transfer and the lack of core synthesis when only one protein is expressed in the cell.

While DALI analysis revealed 2903 to be structurally similar to a number of known glycosyltransferases, the true function of ELM1-like mitochondrial fission proteins remains ill-defined in bacteria (29). It may be that 2903 is not in fact a glycosyltransferase and is instead fulfilling an assistive yet necessary role in core oligosaccharide synthesis. Such a model implies that KdoIII and GlcNAcA are not transferred together and would require one additional glycosyltransferase to fulfill the role of either KdoIII or GlcNAcA transfer with LpsB catalyzing transfer of the other. This model is highly unlikely, however. As demonstrated by our successful partial core reconstitution in *E. coli* WBB06 using *A. baumannii*-derived Gna, 2903, and LpsB, this additional glycosyltransferase would also need to be present in *E. coli* and able to transfer either a third Kdo residue or UDP-GlcNAcA. However, the severely truncated core structure of the *E. coli* WBB06 strain used in our experiment has been reported to prohibit KdoIII modification, and the UDP-GlcNAcA residue is not known to be found in *E. coli* (48, 49). In short, current available data suggest that such a glycosyltransferase is likely not present, supporting the hypothesis that *2903* actually encodes a glycosyltransferase. Indeed, if this is the case, the data reported here will help to elucidate the function of other ELM1-like mitochondrial fission proteins in bacteria.

A question that certainly persists is why *A. baumannii* has evolved such a novel mechanism for core assembly. The shortened, chained morphology and heightened susceptibility to peptidoglycan-targeting antibiotics, particularly in a *2903* mutant, point to a connection between the cell wall and core oligosaccharide assembly (i.e., LOS). Notably, a confirmed mechanism of LOS regulation in *A. baumannii* has not been described. A recent study by Hummels et al. investigating cell envelope biogenesis in *P. aeruginosa* described a specific interaction between the cell wall synthesis enzyme MurA and LpxC (50). LpxC catalyzes the first committed step of LPS/LOS synthesis, and its interaction with MurA promotes coordination of two major cell envelope components, LPS and peptidoglycan. This provides a realistic foundation for a similar yet separate coordination of the cell envelope in *A. baumannii*. While all Gram-negatives must maintain a certain synchronization of the multiple cell envelope components to support cellular rigidity and homeostasis, the fact that *A. baumannii* is able to survive in the absence of LOS—thereby significantly disrupting cell envelope homeostasis—points to a larger phenomenon of speedy and tightly regulated intracellular communication in this organism. Recent studies showing that *A. baumannii* relies heavily on unique aspects of cell wall synthesis in order to survive during LOS deficiency indicate that it would be advantageous to have a tightly controlled feedback mechanism between the cell wall and outer membrane in order to accommodate the extreme disruption in envelope homeostasis (14, 34, 35, 51). The data presented in this study afford early support for such a connection.

Here, we have provided an extensive analysis of the predicted core oligosaccharide locus in *A. baumannii* 17978 that can be extrapolated to a variety of other strains with homology to these described core oligosaccharide synthesis genes. We have provided strong evidence to show that *A. baumannii* utilizes a novel and unusual mechanism of core oligosaccharide synthesis that marks a puzzling departure from the canonical method of Kdo transfer utilized by all other known Gram-negative bacteria. Features of this core oligosaccharide synthesis method, including KdoIII/GlcNAcA transfer and its potential connection to the *A. baumannii* cell wall, provide the basis for further studies probing mechanisms of biogenesis and homeostasis of the Gram-negative cell envelope.

## MATERIALS AND METHODS

### Bacterial strains and growth

Strains and plasmids used can be found in Table S3. Plasmids for gene co-expression (pMMB::*2903*/*lpsB*, pWSKI::*gna*/*2903*/*lpsB*) were synthesized and sub-cloned by GenScript. Cultures were grown in LB medium at 37°C for all experiments. When indicated, culture medium was supplemented with 30 µg/mL kanamycin, 10 µg/mL tetracycline, or 100 µg/mL ampicillin. For all growth curves, cultures were diluted to an OD_600_ of ~0.05 and grown in 5 mL LB broth. A volume of 150 µL was transferred to a 96-well polystyrene microtiter plate each hour, and an OD_600_ measurement was taken using a BioTek Synergy H1 Hybrid microplate reader with Gen5 software. For CFU/mL growth curves, the same cultures were serially diluted in 96-well polystyrene microtiter plates. Five microliters of each dilution was spotted to LB agar plates and incubated overnight. Colonies were counted and used to conduct CFU/mL calculations at each time point. Growth curves were generated using GraphPad Prism.

### Strain construction

All primers used in this study are listed in Table S3. Chromosomal *A. baumannii* deletion mutants were generated using a previously described recombineering method (52). Parent strain cultures carrying an *A. baumannii*-engineered recombineering system (Rec_Ab_) encoded on a plasmid were diluted to an OD_600_ of ~0.05 and grown at 37°C to an OD_600_ of ~0.2. Isopropyl β-D-1-thiogalactopyranoside was added at a concentration of 2 mM, and the culture was incubated again at 37°C to an OD_600_ of ~0.7–0.8. Cells were pelleted and washed with 10% glycerol several times, after which they were mixed with 2 µg of recombineering PCR product and electroporated at 1.8 kV. Outgrowth was performed for 3 hours in 2 mL LB broth + 2 mM IPTG, and recombinants were selected on LB agar + 30 µg/mL kanamycin at 37°C.

To remove kanamycin resistance cassettes, strains were transformed with a plasmid-carrying FLP recombinase machinery via electroporation. Outgrowth was performed at 37°C in 1 mL LB broth for 1 hour, and successful transformants were selected on LB agar + 10 µg/mL tetracycline. Single colonies were passaged on LB agar + 10 µg/mL tetracycline + 1 mM IPTG and incubated at 37°C. Colonies were screened for successful loss of the resistance cassette by kanamycin sensitivity screens and colony PCR confirmation.

### LOS staining

LB broth was inoculated with a single colony and incubated at 37°C overnight. In the morning, culture equivalent to 1 OD_600_ was pelleted, resuspended in 100 µL 1× LDS sample buffer + 5% β-mercaptoethanol, and boiled for 10 minutes. Samples were treated with 1.5 µL proteinase K after returning to room temperature followed by incubation at 55°C overnight. After proteinase K digestion, samples were centrifuged for 3 minutes to separate DNA from polysaccharides. A volume of 10 µL of each sample was loaded onto a 4%–12% Bis-Tris gel in 600 mL MES-SDS running buffer and electrophoresed at 150 V for 1 hour. For more precise separation of whole-cell lysates, samples were resuspended in 100 µL 1× Tricine SDS sample buffer + 5% β-mercaptoethanol and incubated with proteinase K and centrifuged as previously described. A volume of 10 µL of each sample was loaded onto a 16% Tricine gel in 800 mL MES-SDS running buffer and electrophoresed at 125 V for 1.5 hours. Staining with ProQ Emerald 300 was conducted according to the kit manufacturer’s manual.

### LOS purification

LOS was purified via hot water-phenol method as previously described (20). Furthermore, 250 mL LB broth cultures were grown to an OD_600_ of 0.8–1.0 at 37°C. Cells were harvested by centrifugation at 5,000 × *g* for 10 minutes and frozen at −80°C for 24 hours. The bacteria were lyophilized, and the resulting dried sample was resuspended in water to a final concentration of 20 mg/mL. The samples were then transferred to a Teflon centrifuge bottle, mixed with an equal volume of 80% (wt/vol) aqueous phenol, and incubated for 1 hour at 65°C with shaking at 200 rpm. After cooling on ice for 10 minutes, the samples were centrifuged at 5,000 × g for 30 minutes at room temperature, resulting in distinct aqueous and phenol layers. The upper aqueous layer was removed and transferred to a glass conical flask, while the remaining phenol layer was mixed with an equal volume of water. Again, the mixture was incubated at 65°C with shaking, followed by cooling on ice and centrifugation to separate the aqueous and phenol layers. The aqueous layer was removed and combined with the first extraction. The resulting combined aqueous phase was dialyzed against water at 4°C for 24 hours using MWCO 3500 dialysis cassettes. The spent water was exchanged with fresh water six times over the course of dialysis. After 24 hours, the solution was centrifuged at 17,000 × *g* for 20 minutes at room temperature. The supernatant was removed and lyophilized to concentrate the LOS sample. The lyophilized material was resuspended in 10 mL Tris (pH 7.8), mixed with RNase (final concentration 25 µg/mL) and DNase I (final concentration 100 µg/mL), and incubated for 2 hours at 37°C. Proteinase K (final concentration 100 µg/mL) was added, and the solution was incubated again for 2 hours at 37°C. After incubation, 5 mL of water-saturated phenol was added, and the resulting mixture was centrifuged at 5,000 × *g* for 30 minutes at room temperature. The upper aqueous fraction was removed and dialyzed for 12 hours against water at 4°C using MWCO 3500 dialysis cassettes. As before, the spent water was exchanged with fresh water six times. The dialyzed solution was centrifuged at 17,000 × *g* for 20 minutes at room temperature, and the resulting supernatant was lyophilized. Final lyophilized samples were resuspended to a concentration of 5 mg/mL for analysis and stored at −20°C.

### Purified LOS analysis by CID/UVPD mass spectrometry

Intact LOS samples were analyzed using liquid chromatography coupled to ultraviolet photodissociation mass spectrometry as previously described (20, 53). In brief, separations were performed using a Dikma Bio-Bond C8 column (2.1 × 150 mm, 3 µm particle size) (Lake Forest, CA) installed on a Dionex Ultimate 3000 microflow liquid chromatography system (Sunnyvale, CA) using a 40-minute reversed-phase gradient performed at flow rate of 400 µL/min (20). Mobile phase A consisted of 62:36:2 MeOH/H_2_O/CHCl_3_, mobile phase B consisted of 80:20:2 CHCl_3_/MeOH/H_2_O, and mobile phase C consisted of 62:36:2 MeOH/H_2_O/CHCl_3_ with 100 mM ammonium acetate. From 0 to 2 minutes, the solvent composition was held at 15% B and increased to 30% B over 18 minutes. The solvent composition was then held at 30% B for 10 minutes and returned to 15% B for a 10-minute re-equilibration period. Mobile phase C was held at 2%. Ions were generated in the negative ion mode using a heated electrospray ionization source coupled to a Thermo Fisher Scientific Orbitrap Fusion Lumos mass spectrometer modified to perform 193 nm UVPD using a Coherent Excistar XS excimer laser (Santa Clara, CA) (53). Electrospray voltage was set to 4.5 kV, with the sheath gas and auxiliary gases set to 50 and 25 arbitrary units. Each sample was subjected to an initial data-dependent CID survey analysis to screen the mixtures for LOS. The CID mass spectra were manually analyzed to identify the *m/z* values corresponding to the intact lipid A and core oligosaccharide sub-structures of each LOS species detected. Each sample was subsequently subjected to a targeted MS^3^ CID/UVPD experiment to collect fragment spectra for the oligosaccharide moieties. For CID spectra, the normalized collision energy was set to 25. For all UVPD spectra, 10 pulses at 4 mJ per pulse were used. All spectra were manually interpreted with the aid of ChemDraw (PerkinElmer) and labeled using Domon and Costello nomenclature to describe fragment ions (54). Oligosaccharide structures are represented using the official Symbol Nomenclature for Glycans (33).

### Efficiency of plating assays

Overnight cultures were standardized based on OD_600_ measurements and serially diluted in a 96-well plate in LB broth using a multichannel pipette. The cultures were then replica plated onto LB agar media containing no antibiotic, 20 or 25 µg/mL vancomycin, and 5 or 17.5 µg/mL bacitracin and incubated overnight at 37°C.

### Phase contrast microscopy

One microliter of each mid-log phase culture was spotted to 2% agarose pads and visualized using a Nikon Eclipse Ti2 inverted microscope. A minimum of 10 fields of view were captured using NIS-Elements software. Measurements of cell size were performed with MicrobeJ (55) on all fields of view totaling ≥400 cells per strain, and graphs were generated in GraphPad Prism 10. A one-way analysis of variance test was performed with Brown-Forsythe and Welch tests. Games-Howell tests were used to determine statistical significance.

### Isolation of lipid A with truncated core oligosaccharides for MALDI-TOF mass spectrometry

Lipid A-core species from our selective core reconstitution analysis were isolated as previously described (56). Briefly, cells from 100 mL of culture (OD_600_ of ~1.0) were lysed in a single-phase Bligh-Dyer mixture consisting of chloroform/methanol/water (1:2:0.8, vol/vol). Following centrifugation at 2,500 × *g* for 15 minutes, the supernatant which contains lipid A species with highly truncated core oligosaccharides and other lipids (e.g., glycerophospholipids) was transferred to a clean tube. The solution was converted into a two-phase Bligh-Dyer mixture consisting of chloroform/methanol/water (2:2:1.8 vol/vol) and centrifuged again. The lower phase was removed, and the extraction was repeated a second time. The pooled lower phases were converted into a final two-phase Bligh-Dyer, centrifuged, and the lipid-containing lower phases removed to a clean vessel. The samples were dried using a Buchi rotary evaporator and stored at −20°C until further analysis.

### MALDI-TOF mass spectrometry

Lipids were analyzed by matrix-assisted laser desorption ionization-time of flight mass spectrometry in the negative-ion reflectron mode (Bruker Auto-Flex Speed) (57). The matrix used was a saturated solution of 6-aza-2-thiothymine in 50% acetonitrile with saturated tribasic ammonium citrate (20:1, vol/vol). Samples were resuspended in matrix (1:1, vol/vol) and loaded onto the plate as previously described (57).

### Analysis of ^32^P-labeled lipid A

Isolation of ^32^P-labeled lipid A was carried out as previously described (31, 58). To summarize, overnight cultures were diluted to an OD_600_ of 0.05 in 10 mL LB broth containing 5 µCi/mL ^32^P ortho-phosphoric acid (Perkin-Elmer) and grown to an OD_600_ of 1.0. Cells were harvested and washed once with PBS. Lipid A was isolated via Bligh-Dyer extraction following a mild acid hydrolysis that separates the lipid A domain from the core oligosaccharide. Lipid A species were separated by TLC in a chloroform/pyridine/88% formic acid/water (50:50:16:5, vol/vol) solvent system. Plates were dried, exposed to a phosphorimaging screen, and imaged using an Amersham Typhoon laser scanner.

## SUPPLEMENTAL MATERIAL

Supplemental Tables and Figures mbio.03013-23-s0001.docx Tables S1 to S3; Fig. S1 to S5.

ASM does not own the copyrights to Supplemental Material that may be linked to, or accessed through, an article. The authors have granted ASM a non-exclusive, world-wide license to publish the Supplemental Material files. Please contact the corresponding author directly for reuse.
