# Supplementary material for: Characterization of Acinetobacter baumannii core oligosaccharide synthesis reveals novel aspects of lipooligosaccharide assembly
Source: mBio. 2024 Feb 13;15(3):e03013-23. doi: 10.1128/mbio.03013-23 (PMC10936431; doi:10.1128/mbio.03013-23)
Supplement: Supplemental Tables and Figures — Tables S1 to S3; Fig. S1 to S5. [file mbio.03013-23-s0001.docx]

**Supplemental material** supporting “Characterization of *Acinetobacter baumannii* core oligosaccharide synthesis reveals novel aspects of lipooligosaccharide assembly” by VanOtterloo, *et al.*

**Table S1: DALI analysis of 2903**

| **Query** | **Neighbor** | **Z-Score** | **Function** |
| --- | --- | --- | --- |
| 2903 | MurG | 14.8 | Peptidoglycan GlcNAc transferase |
|  | WcaK | 13.9 | Colanic acid pyruvyltransferase |
|  | WaaA | 12.4 | Kdo transferase |
|  | RfaS | 11.7 | Core rhamnosyltransferase |
|  | WaaB | 11.4 | Core galactosyltransferase |
|  | WecB | 11.1 | UDP-N-Acetylglucosamine epimerase (GlcNAc <--> ManNAc) |
|  | LpxB | 10.8 | Lipid A disaccharide synthase |
|  | WaaF | 10.3 | Core HepII transferase |
|  | WcaC | 9.8 | Colanic acid biosynthesis galactosyltransferase |
|  | WecF | 9.7 | ECA N-acetylfucosamine:lipid II N-acetylfucosaminyltransferase |

Analysis was conducted using AF-DB (AlphaFold DataBase) search function of DALI protein structure comparison server. PDB file was downloaded from UniProt database and uploaded to DALI server using *Escherichia coli* as target organism.

**Table S2: PSORTb Analysis of proteins required for *A. baumannii* LOS core assembly.**

| **Protein** | **Predicted localization** |
| --- | --- |
| 2903 | Unknown |
| 2902 | Cytoplasmic |
| 2901 | Unknown |
| 2900 | Cytoplasmic |
| 2899 | Cytoplasmic |
| 2898 | Cytoplasmic |
| 3841 | Cytoplasmic |
| WecB | Cytoplasmic |
| 2896 | Cytoplasmic |
| LpsB | Cytoplasmic |

Amino acid FASTA sequences were submitted to PSORTb version 3.0.3.

| **Table S3: Strains, Plasmids, and Primers used in this study.** | | |
| --- | --- | --- |
| **Strain** | **Genotype** | **Reference** |
| *A. baumannii* 17978 | Wild type 17978 UN, isogenic parent of all 17978 strains used | ATCC |
| *E. coli* W3110 | Wild type, F- λ-, rph-1 IN(*rrnD*, *rrnE*)1 | *E. coli* Genetic Stock Center (Yale) |
| *E. coli* WBB06 | ∆*waaC* ∆*waaF* | (1) |
| *A. baumannii* 17978 | ∆*lpsB* | This study |
| *A. baumannii* 17978 | ∆*2903* | This study |
| *A. baumannii* 17978 | ∆*2902* | This study |
| *A. baumannii* 17978 | ∆*2901* | This study |
| *A. baumannii* 17978 | ∆*2900* | This study |
| *A. baumannii* 17978 | ∆*2899* | This study |
| *A. baumannii* 17978 | ∆*2898* | This study |
| *A. baumannii* 17978 | ∆*3841* | This study |
| *A. baumannii* 17978 | ∆*wecB* | This study |
| *A. baumannii* 17978 | ∆*2896* | This study |
| *A. baumannii* 19606 | Wild type, isogenic parent of all 19606 strains used | ATCC |
| *A. baumannii* 19606 | ∆*lpsB* | This study |
| *A. baumannii* 19606 | ∆*2903* | This study |
| *A. baumannii* 17978 | ∆*lpsB*∆*2903-2899* | This study |
|  |  |  |
| **Plasmid** | **Description** | **Reference** |
| pKD4 | FRT-flanked KanR cassette used for recombineering | (2) |
| pMMBtet-RecAb | pMMB67EH with *A. baumannii* recombineering system and TetR cassette | (3) |
| pMMBtet-FLP | pMMB67EH with FLP recombinase and TetR cassette | (3) |
| pMMB | pMMB67EH with KanR cassette | (3) |
| pMMB::*2903* | pMMB67EH with *2903* | This study |
| pMMB::*lpsB* | pMMB67EH with *lpsB* | This study |
| pMMB::*2903/lpsB* | pMMB67EH with *2903* and *lpsB*. Construct was synthesized by Genscript | This study, GenScript |
| pWSKI | pWSK29 backbone with additional *lacI* and promoter from pET28 | (4) |
| pWSKI::*gna* | pWSKI with *gna* | This study |
| pWSKI::*2903* | pWSKI with *2903* | This study |
| pWSKI::*lpsB* | pWSKI with *lpsB* | This study |
| pWSKI::*gna/2903/lpsB* | pWSKI with *gna, 2903*, and *lpsB*. Construct was synthesized by GenScript | This study, GenScript |
|  |  |  |
| **Primer** | **Sequence (5'→3')** | **Purpose** |
| delta-lpsB-kan-F | TTGTAACACAACGCAACAGCGACATCCATGTCGCTAGCACGATAGTAAACTCCGGATAATGATTTAGTTTGAGATGATGTATCTATGGATGATCCATCTTTTATGAACGTATAAGGCAAAAAACAgcgattgtgtaggctggagctgcttcg | Recombineering in 17978 |
| delta-lpsB-kan-R | AGTAAGTGGGAAAAACAGTACGACTATGAAAAAGCTGCTGCTTATGAAGCATCTCTTAAGTAATAAAGAAGTTTTTTAGTCGAAAAAAACCCGCGAACATCGCGGGTTTTTTTTATTGGGTTATTatatcctccttagttcctattccg | Recombineering in 17978 |
| delta-2903-kan-F | TGATGCAGTTCAAGGTAAAAATCCAAAATATGAACACTGGTTAACTTACGTAAAATAAGTTAATCCGTTAACATGAAAAGGCGAACGTTGGTTTCGCCTTTTTATTTTTGGGTTAGGGAGCTTTTgcgattgtgtaggctggagctgcttcg | Recombineering in 17978 |
| delta-2903-kan-R | AATGGCCATTTTCAATTAATCCGGCAGAAATTTTACGGTCTGTGCAGTAAAAATGTGCGCCTTGTTTATTAAAGCCAAAATTTGCAATATGCAATATTTTCATTTATTTACTCAAAATAAAAAATatatcctccttagttcctattccg | Recombineering in 17978 |
| delta-2902-kan-F | ATCACAACTAATTTCACAGAATACTTGTGTCGTACAGTTGCCACAACCTTATGCTTTTAAAGAAGCAGACCGTGTGGCAACATATCTTTTAGCGAAAAATtaaATTTTTTATTTTGAGTAAATAAgcgattgtgtaggctggagctgcttcg | Recombineering in 17978 |
| delta-2902-kan-R | CTGATGTAACATCACAATGCGAGGTAGTTTACTCGAACGAAACGGAAGCCAGAATGTATAATTACCCAGCACATATAACAGTGCAGCAAGTGCTAGCAGGCCCACCGCAACGTACAAAAACCACAatatcctccttagttcctattccg | Recombineering in 17978 |
| delta-2901-kan-F | CGTAAATCTTTCAATGCTAAGCGTATCACCCAATTTATGGTTGAGGTGACGTTTAAACAGCCCCTTTCAGAAGATTATGAATGGTCACATGAGGTTTATGCATGATGTGGTTTTTGTACGTTGCGgcgattgtgtaggctggagctgcttcg | Recombineering in 17978 |
| delta-2901-kan-R | AGTATTATCAGTACTGCCAGAGTCTAAAATAATGACTTCATCAAACTCACTGACACTTTGTAAAGTTCGCTCTAACCAAGCGCCACAGTTTAAAGTGACAATATAAACACTACATGGAATTTTATatatcctccttagttcctattccg | Recombineering in 17978 |
| delta-2900-kan-F | TCGACTCGTAAAAAAATCGAAGCGTATGAAGCAGATAACCAATTTAATATTCCACGTGTCAGCACGCATGGCGCTATGAATGCTTTACAGATGCGTATCGCCCTTGCTAAAGGACGTTATAAGTTgcgattgtgtaggctggagctgcttcg | Recombineering in 17978 |
| delta-2900-kan-R | AAGGATGTGCAATCACATGTACTTCATGGTTTAAAGCAAGCTCATTAACAAGGTTAAAAGTGTGCTGCTCTAATCCACCAACTCCACCGCTAGTTGCCAATACCTGAACAATTTTCATAGCTTGCatatcctccttagttcctattccg | Recombineering in 17978 |
| delta-2899-kan-F | CCGTAGTTTCTTAAATGGTTGGCGTGGTTTCGTAAATAGTATGATTAATGCATTTTATGCATTTCTAAAAGAAGCCAAACTATTTGAACAGACGATGAATAAAGATAAAAATAAGTAGGCAAGCTgcgattgtgtaggctggagctgcttcg | Recombineering in 17978 |
| delta-2899-kan-R | TATGAAGAAAAATTACTAGAAGTAGTTTTACCAGATTGGAATCAAGCAGATGACGGATGCCATACTATTAATGTCGAAGATAATTTCGTGGTACTTGATGCCATCCGCCTTACACCAAAAAATAAatatcctccttagttcctattccg | Recombineering in 17978 |
| delta-2898-kan-F | TTAACAAAATGAAAAATGATTTAATTTCTCGGGTTATTTCTCTTTTTATTTTCAAAAAAATTATTCGATTAGCATACTTTCTCCAATCCGCCCAAACACAAAAATGAGTAGCTACAGGTAATGAAgcgattgtgtaggctggagctgcttcg | Recombineering in 17978 |
| delta-2898-kan-R | GAATATGCCCTTCAACCAGAGGCTCAAAATGACTTTGGCCCTTTGTATGCAAAAGCCAAGGCTGAACTGACTGTACCAGCCATGGCTGAGCAGACGCTTAACATTTATAAAGGCCTGCTGAAAACatatcctccttagttcctattccg | Recombineering in 17978 |
| delta-3841-kan-F | GCAAAATCTAAAATATTAATCAGATTATTGCGTAAATGAGCGTTTTGTATGATATCGCGAGGCTATCAACAATAAGCATAGCAGGCTAAAATAGCTAGAATGTATTGTATCAAGTTAGTTGAAACgcgattgtgtaggctggagctgcttcg | Recombineering in 17978 |
| delta-3841-kan-R | TGCTCTAACCAGCCAAAGCGCATATACCAATGAGAGTCAAAAGTTTTTGCTTTTTTTGCTTGTTGGTACTTGTACCATTTTTTTTGAAGACCGCGAGTTATTAACATTTCATTACCTGTAGCTACatatcctccttagttcctattccg | Recombineering in 17978 |
| delta-wecB-kan-F | TGTTGATAGCCTCGCGATATCATACAAAACGCTCATTTACGCAATAATCTGATTAATATTTTAGATTTTGCATGTTCTGTTTATTTATTCTGAACTATCTAGAAAAAGGAATAATTTACTTCATTgcgattgtgtaggctggagctgcttcg | Recombineering in 17978 |
| delta-wecB-kan-R | ACAATTAGAATCTTTAGAAAATATTACTCACAGTAAAAACTTTCTAAAGACTCTAGCTACTCGTATTAAAATTAAAGATTTATTTAAACTTATACGTTATTTTATACAATATAAACTAAAGTAATatatcctccttagttcctattccg | Recombineering in 17978 |
| delta-2896-kan-F | TCGGTGCATTACTTATCTTTGTTGCACTGATTTATATGGTGCTCAAAGGTAATGCGGATAAATAAAATATCTACGCTACAGTTTCTTTATTTTTAAATATTATTTTGAAAGACACACAAGATTATgcgattgtgtaggctggagctgcttcg | Recombineering in 17978 |
| delta-2896-kan-R | AACATTATTGGAAAATAGTGAGCTGTATCATCAGATGGCTAGAGCTAACAACCCTTATGGGGATGGTACAGCTAGTATTAAAATCAAACAAATTCTTTTGGATAAATATAGTCATACAATATAAAatatcctccttagttcctattccg | Recombineering in 17978 |
| 19delta-lpsB-kan-F | TTGTAACACAACGCAACAGCGACATCCATGTCGCTATCACGATAGTAAACTCTGGATAATGATTTAGTTTGAGATGATGTATCTATGGATGATCCATCTTTTATGAACGTATAAGGCAAAAAATAgcgattgtgtaggctggagctgcttcg | Recombineering in 19606 |
| 19delta-lpsB-kan-R | GTAATCAGTAAATGGGAAAAACAATACGACTATGAAAAAGCTGCTGCTTATGAAGCATCTCTTAAGTAATAGAGAAGTTTTTTAGTCGAAAAAACCCGCGAACATCGCGGGTTTTTTTTATTGGGatatcctccttagttcctattccg | Recombineering in 19606 |
| 19delta-2903-kan-F | TGATGCAGTTCAAGGTAAAAATCCAAAATATGAACACTGGTTAACTTACGTAAAATAAGTTAATCCGTTAACATGAAAAGGCGAACGTTGGTTTCGCCTTTTTATTTTTGGGTTAGGGAGCTTTTgcgattgtgtaggctggagctgcttcg | Recombineering in 19606 |
| 19delta-2903-kan-R | AATGGCCATTTTCAATTAATCCGGCAGAAATTTTACGGTCTGTGCAGTAAAAATGTGCGCCTTGTTTATTAAAGCCAAAATTTGCAATATGCAATATTTTCATTTATTTACTCAAAATAAAAAATatatcctccttagttcctattccg | Recombineering in 19606 |
| lpsB-screen-F | CAACTCAATATATGTGGTTTCACCGTCG | Chromosomal allele screening |
| lpsB-screen-R | CGTTGAACGTTGTAGGTAACGCGC | Chromosomal allele screening |
| 2903-screen-F | CCACTGAAATCCAAAAGAC | Chromosomal allele screening |
| 2903-screen-R | CGGAAACTAAAATCATAAACG | Chromosomal allele screening |
| 2902-screen-F | GTTGTCGCGTAGGGGTCATAGCA | Chromosomal allele screening |
| 2902-screen-R | AACAGTGCAGCAAGTGCTAGCAG | Chromosomal allele screening |
| 2901-screen-F | GAGCAGGAAGTGGTTTACTTTGATGATCAG | Chromosomal allele screening |
| 2901-screen-R | CCCTGCCAGTCTTGGTGAGAAATTTG | Chromosomal allele screening |
| 2900-screen-F | AGAGATTGGCTTTAAAAATGC | Chromosomal allele screening |
| 2900-screen-R | TGGCTAAACTTGTCTACATAG | Chromosomal allele screening |
| 2899-screen-F | CTTTTATGAAGTCTTATTTTATTC | Chromosomal allele screening |
| 2899-screen-R | TACTCAGCTTAGTCCAGCAG | Chromosomal allele screening |
| 2898-screen-F | ATTGCTTTGAGCATGACGACGAGATG | Chromosomal allele screening |
| 2898-screen-R | GGTACAGTCAGTTCAGCCTTGGCT | Chromosomal allele screening |
| 3841-screen-F | GCCATTTTAATAGCTTCAGGACGCGTAC | Chromosomal allele screening |
| 3841-screen-R | CCGGATGTTGATCATCCACCTCTTCA | Chromosomal allele screening |
| wecB-screen-F | TGCTTAGACTTACCTAAAAATTTTGCTACGCATC | Chromosomal allele screening |
| wecB-screen-R | GACCGATTGCAAACTTTGATGTTGCTG | Chromosomal allele screening |
| 2896-screen-F | TAGTCGTGATTGTATTCTCCCTCGCAC | Chromosomal allele screening |
| 2896-screen-R | CAAGAAGCTATTGATGCAGGCACAGTC | Chromosomal allele screening |
| 19-lpsB-screen-F | CGGATAGGCAGACCTTACTTTTTAGGGATAAAA | Chromosomal allele screening |
| 19-lpsB-screen-R | CGTTGAACGTTGTAGGTAACGCGC | Chromosomal allele screening |
| 19-2903-screen-F | TAACACCAATTCGTGAATACGATGACCG | Chromosomal allele screening |
| 19-2903-screen-R | CATCTGGACTCATCAAGTCGCCATG | Chromosomal allele screening |
| EcoRI-lpsB-F | TAGCATGAATTCatgAAAGTGATGCAACTTCTCCCAGAAC | Plasmid cloning (pMMB) |
| BamHI-lpsB-R | TAGCATGGATTCTCAATTCAATACACTTTGATATAGCTCAAGGGTTTGG | Plasmid cloning (pMMB) |
| EcoRI-2903-F | TAGCATGAATTCATGCATATTGTCTATGTCTCTGATGGTAAAGCAG | Plasmid cloning (pMMB) |
| BamHI-2903-R | TAGCATGGATCCTTAATTTTTCGCTAAAAGATATGTTGCCACACGG | Plasmid cloning (pMMB) |
| SacI-gna-F | TAGCATgagctcAGGAGGCTTTTatgCAACTTGCCGATTTAAGAATAGCGA | Plasmid cloning (pWSKI) |
| BamHI-gna-R | TAGCATggatccttaGAGACGGAATACAGTAAAACCAACATCTGAC | Plasmid cloning (pWSKI) |
| SacI-RBSEc-lpsB-F | TAGCATGAGCTCAGGAGGAAACAATGAAAGTGATGCAACTTCTCCCAG | Plasmid cloning (pWSKI) |
| BamHI-lpsB-R | TAGCATGGATTCTCAATTCAATACACTTTGATATAGCTCAAGGGTTTGG | Plasmid cloning (pWSKI) |
| SacI-RBSEc-2903-F | TAGCATGAGCTCAGGAGGCTTTTATGCATATTGTCTATGTCTCTGATGGTAAAGCA | Plasmid cloning (pWSKI) |
| BamHI-2903-R | TAGCATGGATCCTTAATTTTTCGCTAAAAGATATGTTGCCACACGG | Plasmid cloning (pWSKI) |

**References (Supplemental)**

1. Brabetz W, Müller-Loennies S, Holst O, Brade H. 1997. Deletion of the heptosyltransferase genes *rfaC* and *rfaF* in *Escherichia* *coli* K-12 results in an Re-type lipopolysaccharide with a high degree of 2-aminoethanol phosphate substitution. Eur J Biochem 247:716–724.

2. Datsenko KA, Wanner BL. 2000. One-step inactivation of chromosomal genes in *Escherichia* *coli* K-12 using PCR products. PNAS 97:6640–6645.

3. Tucker AT, Nowicki EM, Boll JM, Knauf GA, Burdis NC, Trent MS, Davies BW. 2014. Defining Gene-Phenotype Relationships in *Acinetobacter* *baumannii* through One-Step Chromosomal Gene Inactivation. mBio 5:e01313-14.

4. Purcell AB, Simpson BW, Trent MS. 2023. Impact of the cAMP-cAMP Receptor Protein Regulatory Complex on Lipopolysaccharide Modifications and Polymyxin B Resistance in *Escherichia* *coli*. Journal of Bacteriology 205:e00067-23.

**
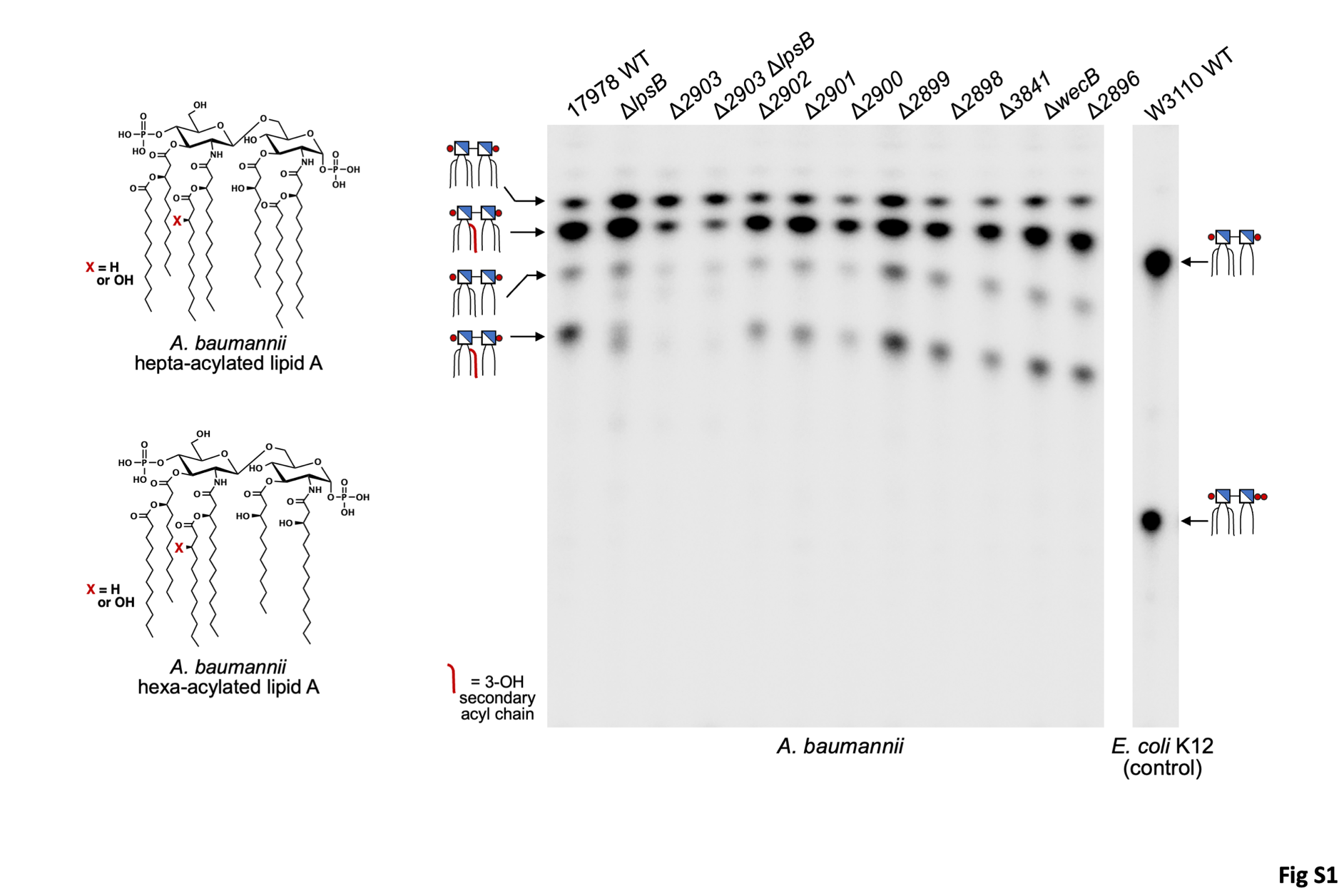
**

**Figure S1: Lipid A analysis of *A. baumannii* core truncation mutants.** ^32^P-labeled lipid A from the indicated *A. baumannii* strains was isolated, separated by TLC, and visualized by phosphorimaging. *E. coli* K-12 strain W3110, producing two major lipid A species, was included as a control. The observed lipid A species are depicted as cartoons for ease of interpretation. Also, the chemical structures of both *A. baumannii* hexa-acylated and hepta-acylated lipid A are shown.

**
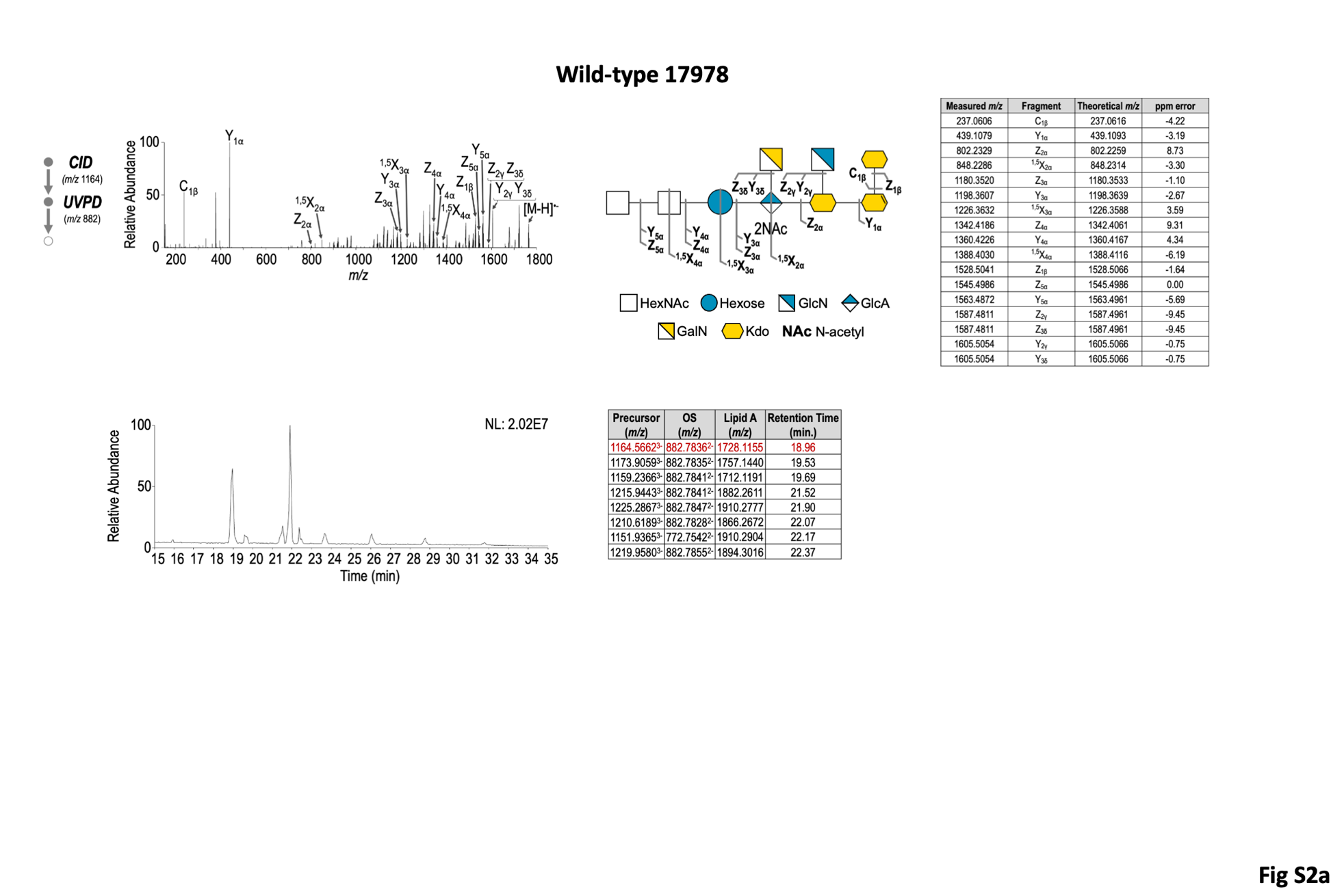
**

**Figure S2a: MS^3^ analysis of core oligosaccharide from *A. baumannii* 17978*.*** CID of the precursor ion of *m/z* 1164.57 (z=3) followed by UVPD of the fragment ion of *m/z* 882.79 (z=2) where z indicates the charge state. To the right of the spectrum is an oligosaccharide fragment ion map and a list of identified fragment ions with calculated ppm error. Below the spectrum is a chromatogram listing all identified LOS species based on CID MS^2^ spectra. Oligosaccharide fragment ion maps are depicted using the official Symbol Nomenclature for Glycans (SNFG) and Domon and Costello carbohydrate fragmentation nomenclature. H_2_O loss from Kdo upon cleavage of the glycosidic bond between the core oligosaccharide and lipid A during CID is depicted as a double bond on the Kdo symbol.


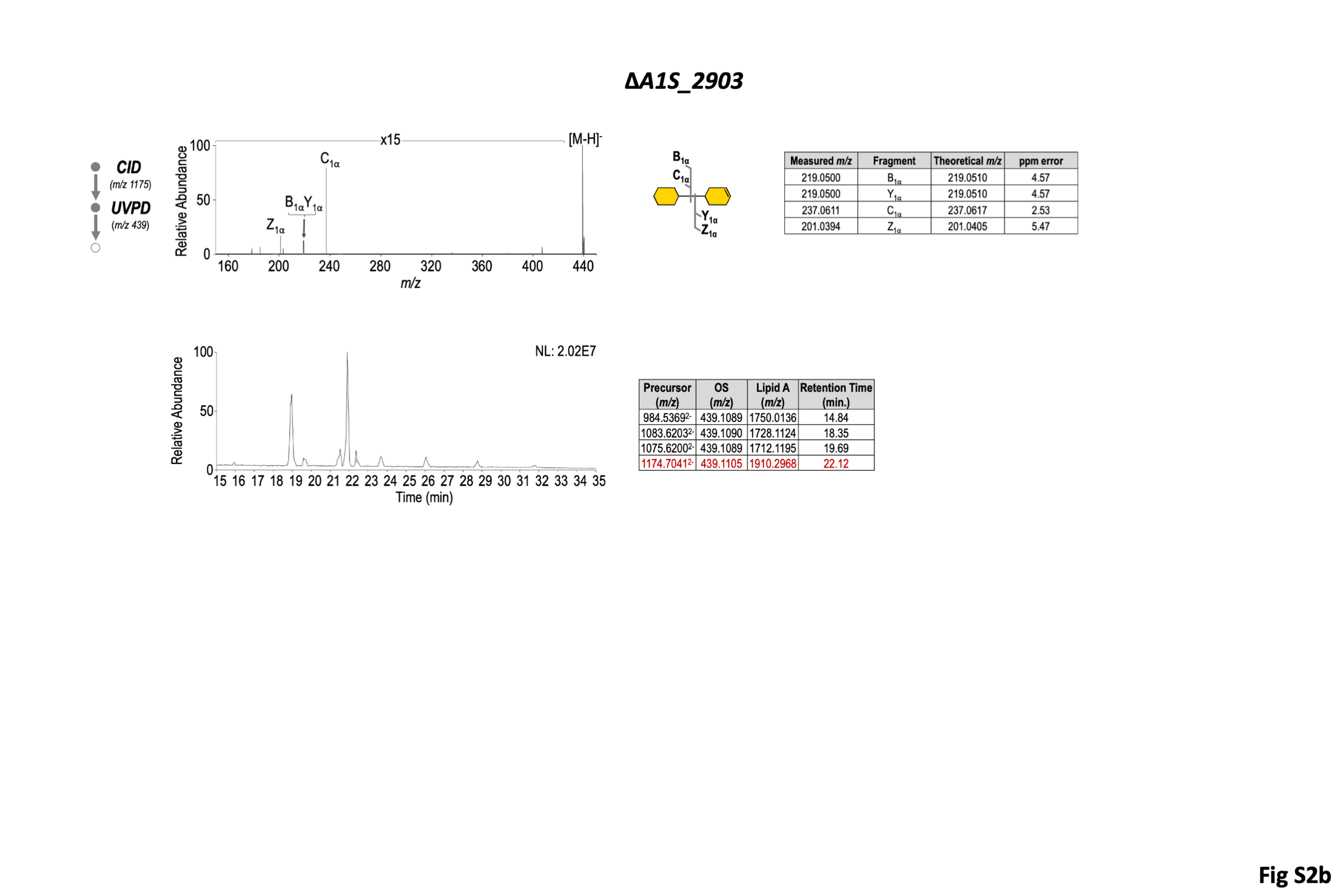


**Figure S2b: MS^3^ analysis of core oligosaccharide from *A. baumannii* Δ*2903.*** CID of the precursor ion of *m/z* 1174.7041 (z=2) followed by UVPD of the fragment ion of *m/z* 439.1105 (z=1). To the right of the spectrum is an oligosaccharide fragment ion map and a list of identified fragment ions with calculated ppm error. Below the spectrum is a chromatogram with a list of all identified LOS species based on CID MS^2^ spectra. Oligosaccharide fragment ion maps are depicted using the official Symbol Nomenclature for Glycans (SNFG) and Domon and Costello carbohydrate fragmentation nomenclature. H_2_O loss from Kdo upon cleavage of the glycosidic bond between the core oligosaccharide and lipid A during CID is depicted as a double bond on the Kdo symbol.

**
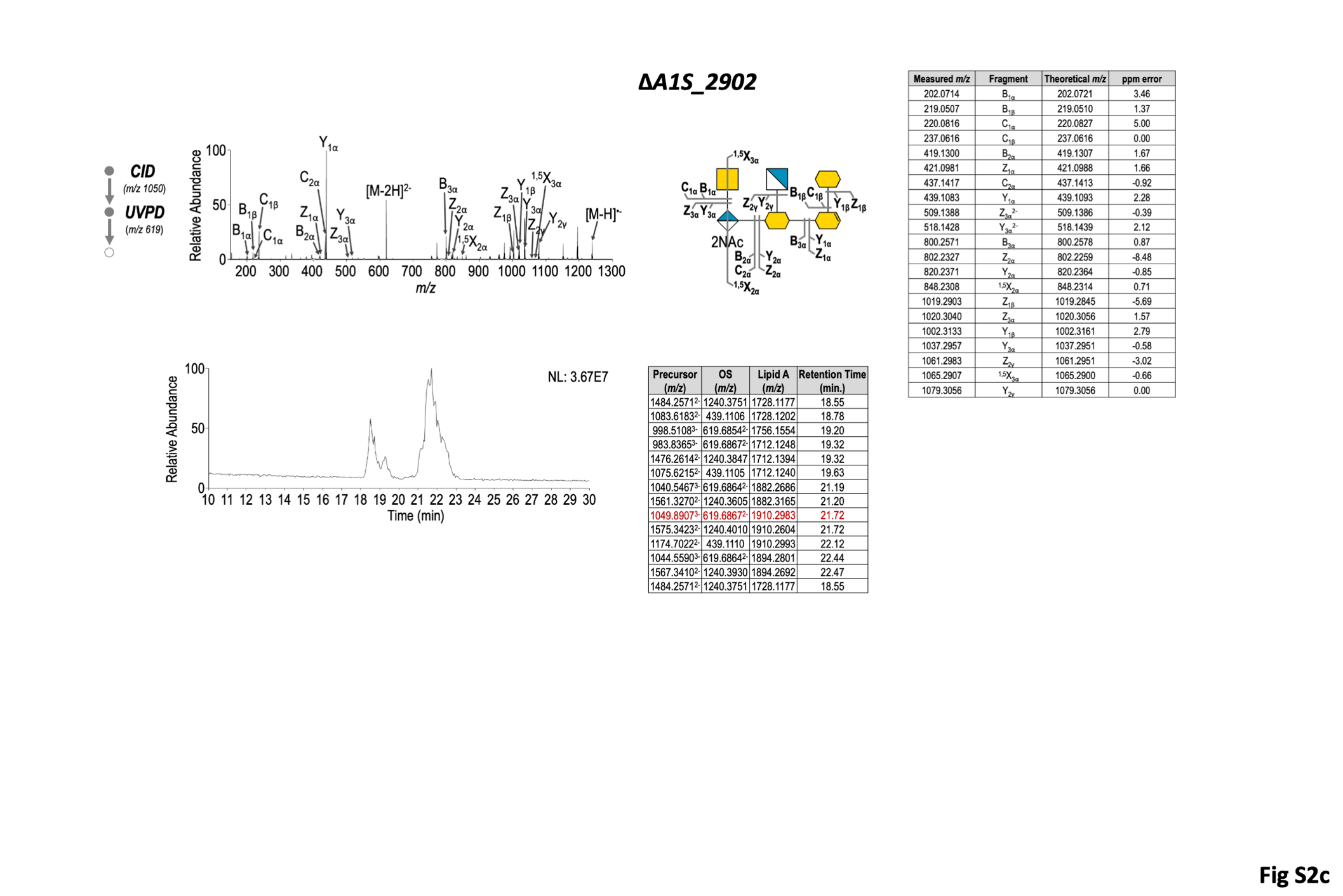
**

**Figure S2c: MS^3^ analysis of core oligosaccharide from *A. baumannii ∆2902.*** CID of the precursor ion of *m/z* 1049.89 (z=3) followed by UVPD of the fragment ion of *m/z* 619.69 (z=2). To the right of the spectrum is an oligosaccharide fragment ion map and a list of identified fragment ions with calculated ppm error. Below the spectrum is a chromatogram with a list of all identified LOS species based on CID MS^2^ spectra. Oligosaccharide fragment ion maps are depicted using the official Symbol Nomenclature for Glycans (SNFG) and Domon and Costello carbohydrate fragmentation nomenclature. H_2_O loss from Kdo upon cleavage of the glycosidic bond between the core oligosaccharide and lipid A during CID is depicted as a double bond on the Kdo symbol.


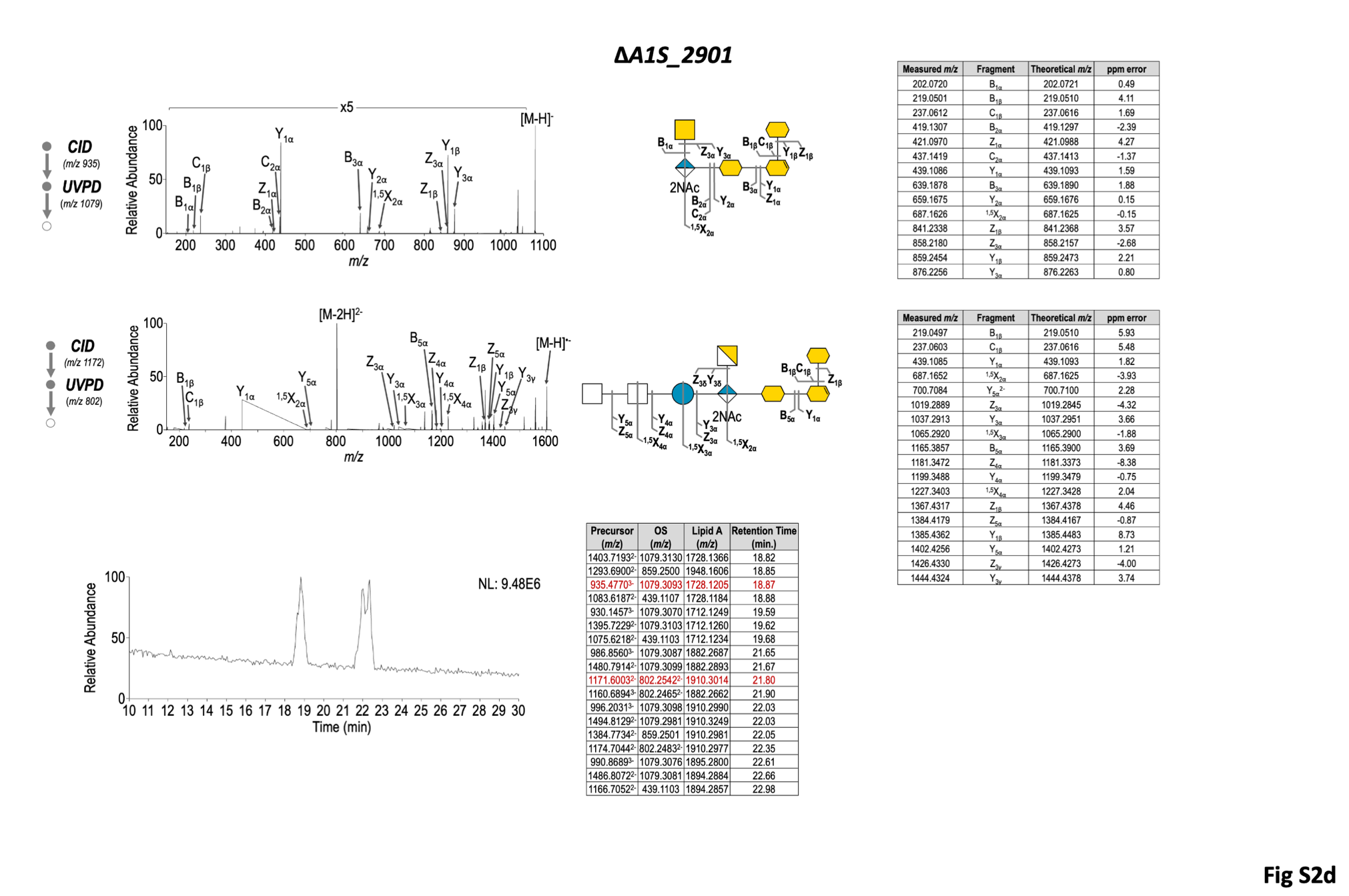


**Figure S2d: MS^3^ analysis of core oligosaccharide from *A. baumannii ∆2901.* Upper panel:** CID of the precursor ion of *m/z* 935.48 (z=3) followed by UVPD of the fragment ion of *m/z* 1079.31 (z=1). **Middle panel:** CID of the precursor ion of *m/z* 1171.60 (z=3) followed by UVPD of the fragment ion of *m/z* 802.25 (z=2). To the right of the spectra are oligosaccharide fragment ion maps and lists of identified fragment ions with calculated ppm error. Below the pair of spectra is a chromatogram with a list of all identified LOS species based on CID MS^2^ spectra. Oligosaccharide fragment ion maps are depicted using the official Symbol Nomenclature for Glycans (SNFG) and Domon and Costello carbohydrate fragmentation nomenclature. H_2_O loss from Kdo upon cleavage of the glycosidic bond between the core oligosaccharide and lipid A during CID is depicted as a double bond on the Kdo symbol.


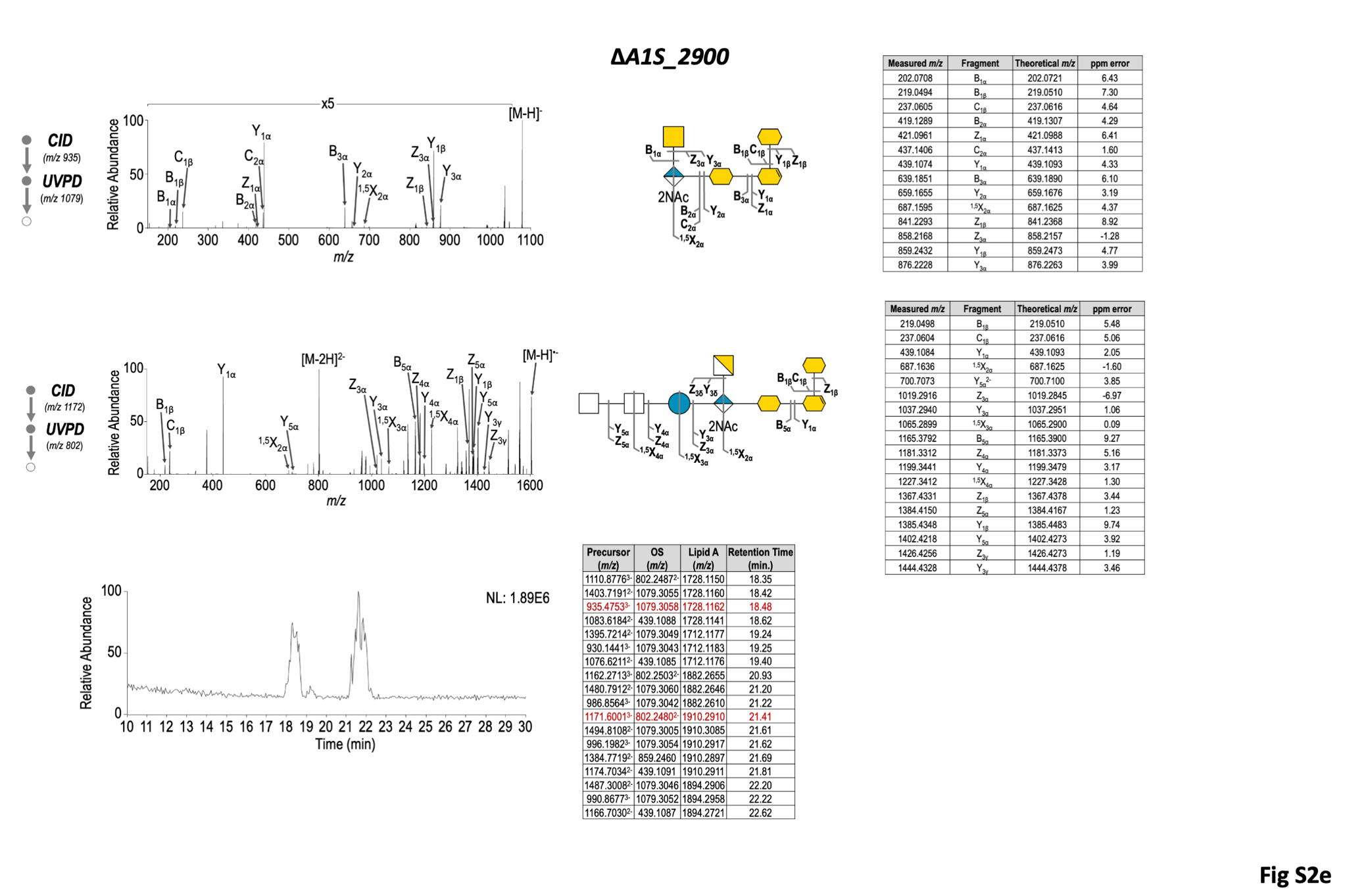


**Figure S2e: MS^3^ analysis of core oligosaccharide from *A. baumannii ∆2900.* Upper panel:** CID of the precursor ion of *m/z* 935.45 (z=3) followed by UVPD of the fragment ion of *m/z* 1079.31 (z=1). **Middle panel:** CID of the precursor ion of *m/z* 1171.60 (z=3) followed by UVPD of the fragment ion of *m/z* 802.25 (z=2). To the right of the spectra are oligosaccharide fragment ion maps and lists of identified fragment ions with calculated ppm error. Below the spectra is a chromatogram with a list of all identified LOS species based on CID MS^2^ spectra. Oligosaccharide fragment ion maps are depicted using the official Symbol Nomenclature for Glycans (SNFG) and Domon and Costello carbohydrate fragmentation nomenclature. H_2_O loss from Kdo upon cleavage of the glycosidic bond between the core oligosaccharide and lipid A during CID is depicted as a double bond on the Kdo symbol.


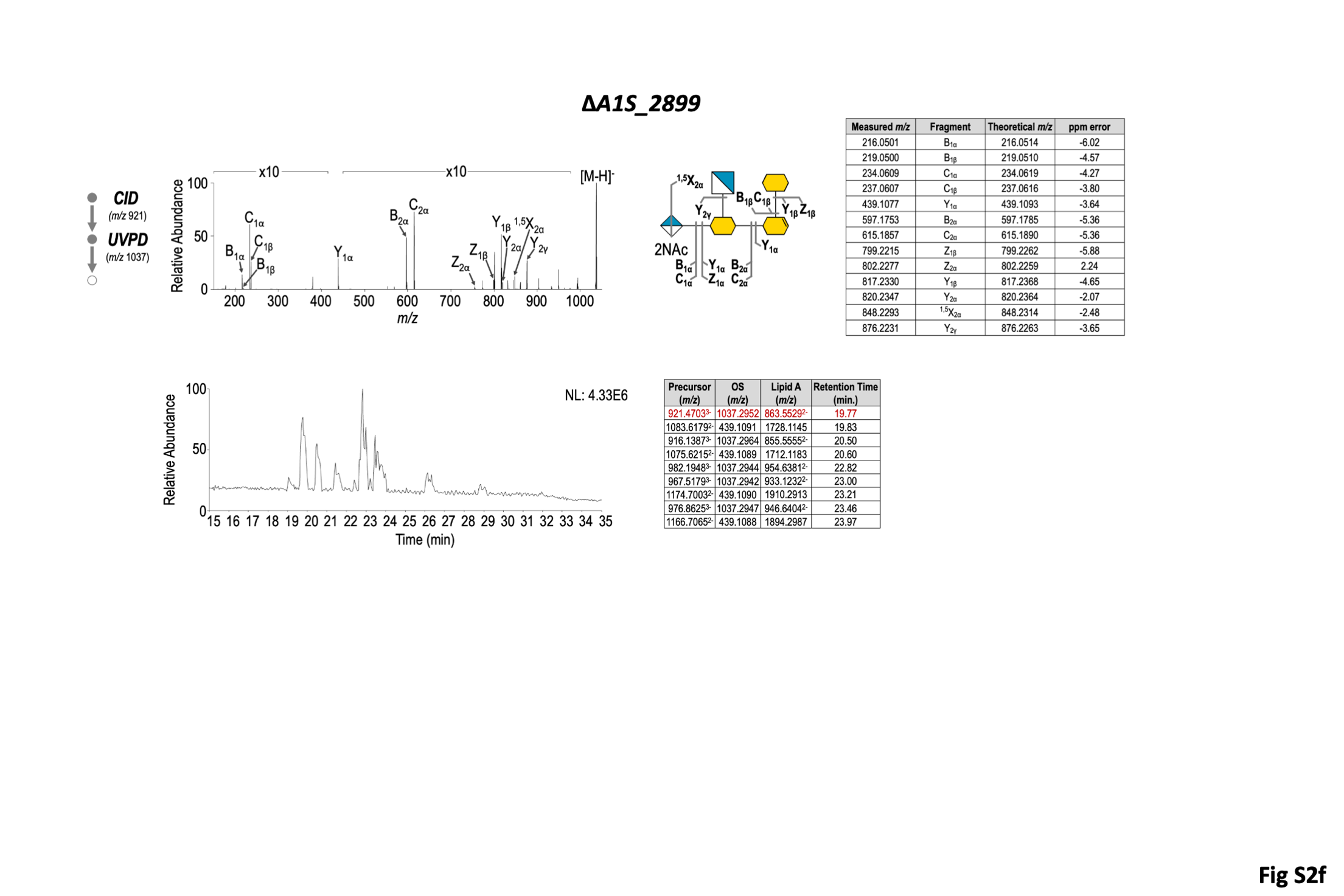


**Figure S2f: MS^3^ analysis of core oligosaccharide from *A. baumannii ∆2899.*** CID of the precursor ion of *m/z* 921.47 (z=3) followed by UVPD of the fragment ion of *m/z* 1037.29 (z=1). To the right of the spectrum is an oligosaccharide fragment ion map and a list of identified fragment ions with calculated ppm error. Below the spectrum is a chromatogram with a list of all identified LOS species based on CID MS^2^ spectra. Oligosaccharide fragment ion maps are depicted using the official Symbol Nomenclature for Glycans (SNFG) and Domon and Costello carbohydrate fragmentation nomenclature. H_2_O loss from Kdo upon cleavage of the glycosidic bond between the core oligosaccharide and lipid A during CID is depicted as a double bond on the Kdo symbol.

**
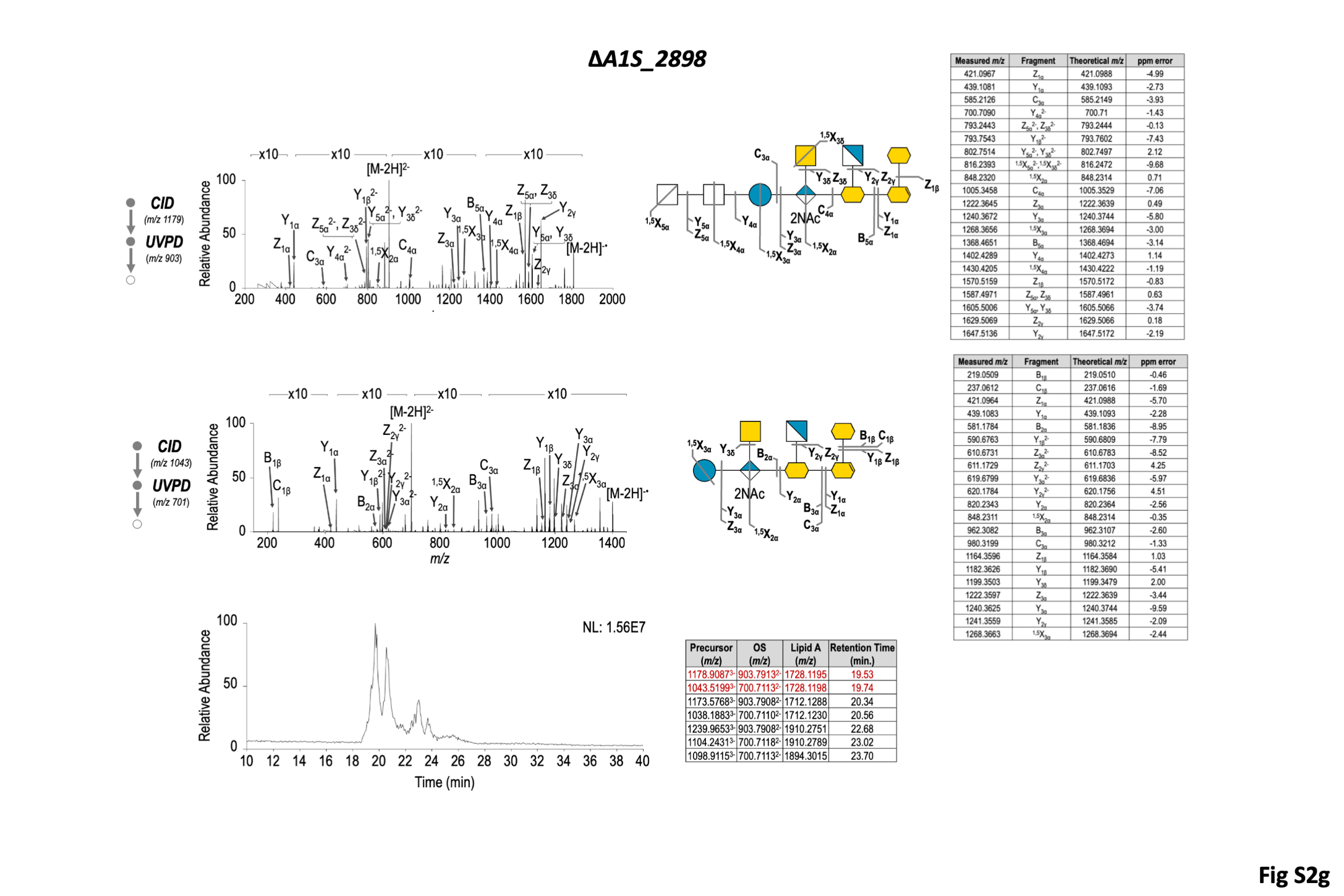
**

**Figure S2g: MS^3^ analysis of core oligosaccharide from *A. baumannii ∆2898.* Upper panel:** CID of the precursor ion of *m/z* 1178.9082 (z=3) followed by UVPD of the fragment ion of *m/z* 903.7919 (z=2). **Middle panel:** CID of the precursor ion of *m/z* 1043.5199 (z=3) followed by UVPD of the fragment ion of *m/z* 700.7113 (z=2). To the right of the spectra are oligosaccharide fragment ion maps and lists of identified fragment ions with calculated ppm error. Below the spectra is a chromatogram with a list of all identified LOS species based on CID MS^2^ spectra. Oligosaccharide fragment ion maps are depicted using the official Symbol Nomenclature for Glycans (SNFG) and Domon and Costello carbohydrate fragmentation nomenclature. H_2_O loss from Kdo upon cleavage of the glycosidic bond between the core oligosaccharide and lipid A during CID is depicted as a double bond on the Kdo symbol.


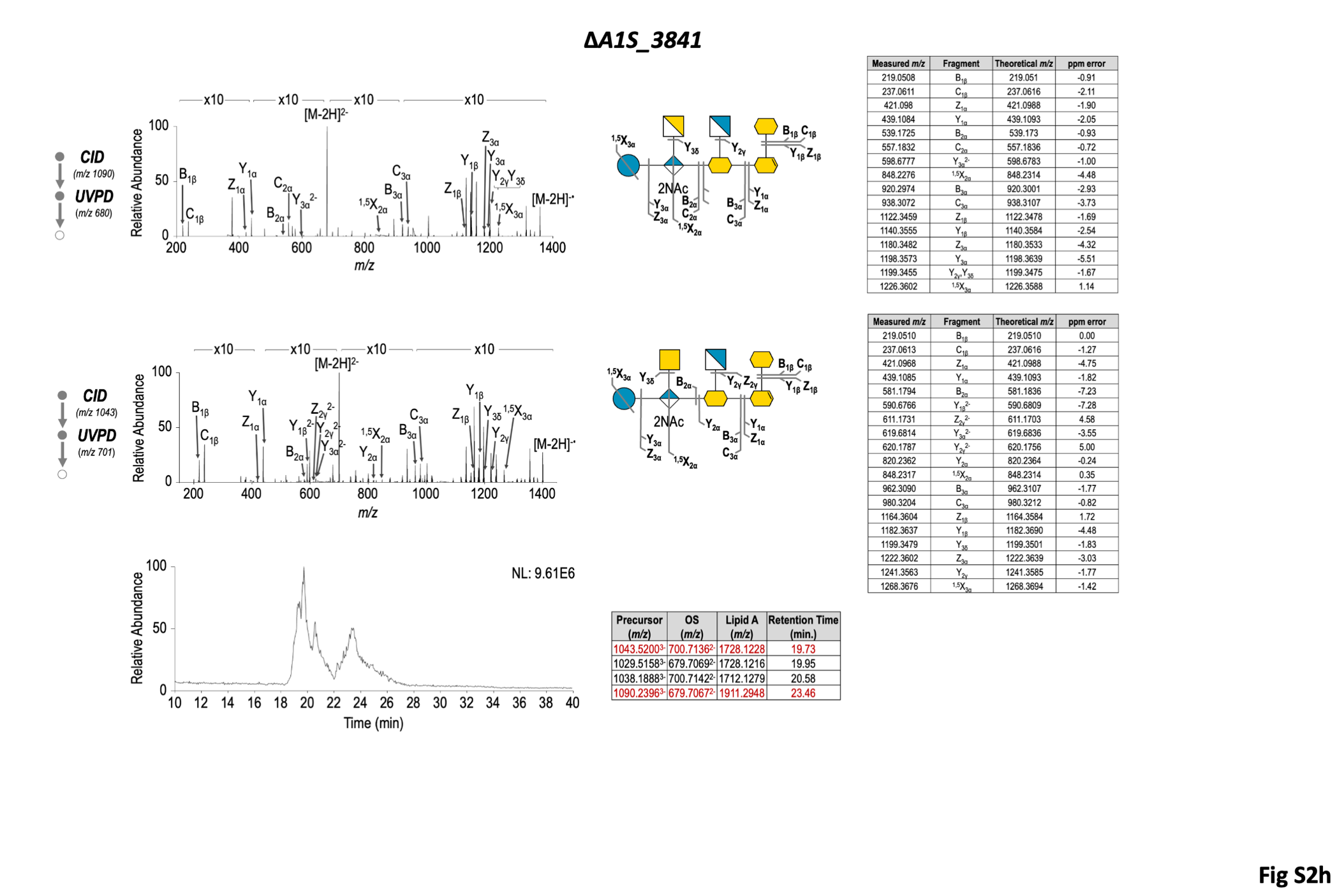


**Figure S2h: MS^3^ analysis of core oligosaccharide from *A. baumannii ∆3841.* Top:** CID of the precursor ion of *m/z* 1090.2356 (z=3) followed by UVPD of the fragment ion of *m/z* 679.7067 (z=2). **Bottom:** CID of the precursor ion of *m/z* 1043.5202 (z=3) followed by UVPD of the fragment ion of *m/z* 700.7130 (z=2). To the right of the spectra are oligosaccharide fragment ion maps and lists of identified fragment ions with calculated ppm error. Below the spectra is a chromatogram with a list of all identified LOS species based on CID MS^2^ spectra. Oligosaccharide fragment ion maps are depicted using the official Symbol Nomenclature for Glycans (SNFG) and Domon and Costello carbohydrate fragmentation nomenclature. H_2_O loss from Kdo upon cleavage of the glycosidic bond between the core oligosaccharide and lipid A during CID is depicted as a double bond on the Kdo symbol.


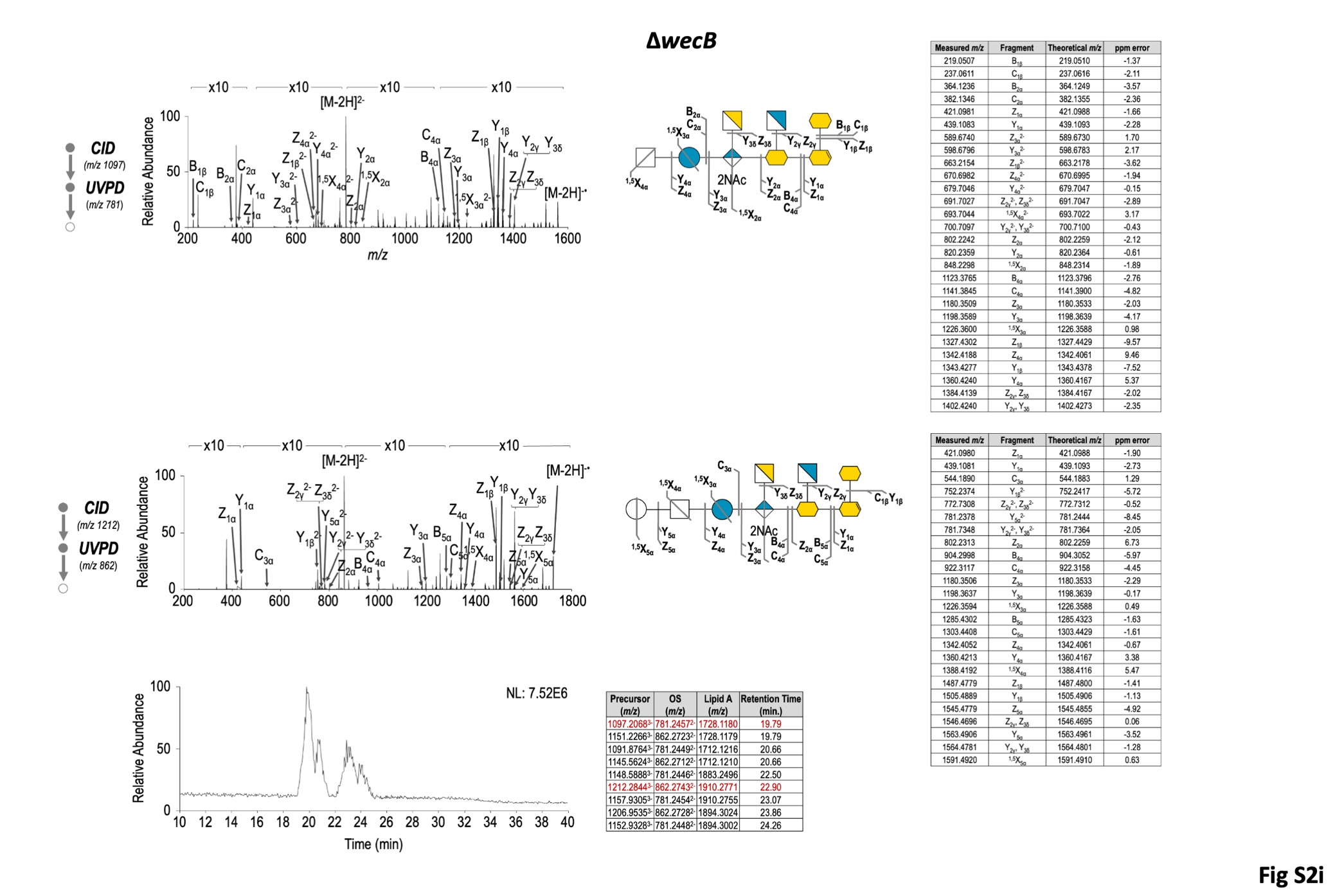


**Figure S2i: MS^3^ analysis of core oligosaccharide from *A. baumannii ∆wecB.* Top:** CID of the precursor ion of *m/z* 1097.2121 (z=3) followed by UVPD of the fragment ion of *m/z* 781.2480 (z=2). **Bottom:** CID of the precursor ion of *m/z* 1212.2844 (z=3) followed by UVPD of the fragment ion of *m/z* 862.2743 (z=2). To the right of the spectra are oligosaccharide fragment ion maps and lists of identified fragment ions with calculated ppm error. Below the spectra is a chromatogram with a list of all identified LOS species based on CID MS^2^ spectra. Oligosaccharide fragment ion maps are depicted using the official Symbol Nomenclature for Glycans (SNFG) and Domon and Costello carbohydrate fragmentation nomenclature. H_2_O loss from Kdo upon cleavage of the glycosidic bond between the core oligosaccharide and lipid A during CID is depicted as a double bond on the Kdo symbol.


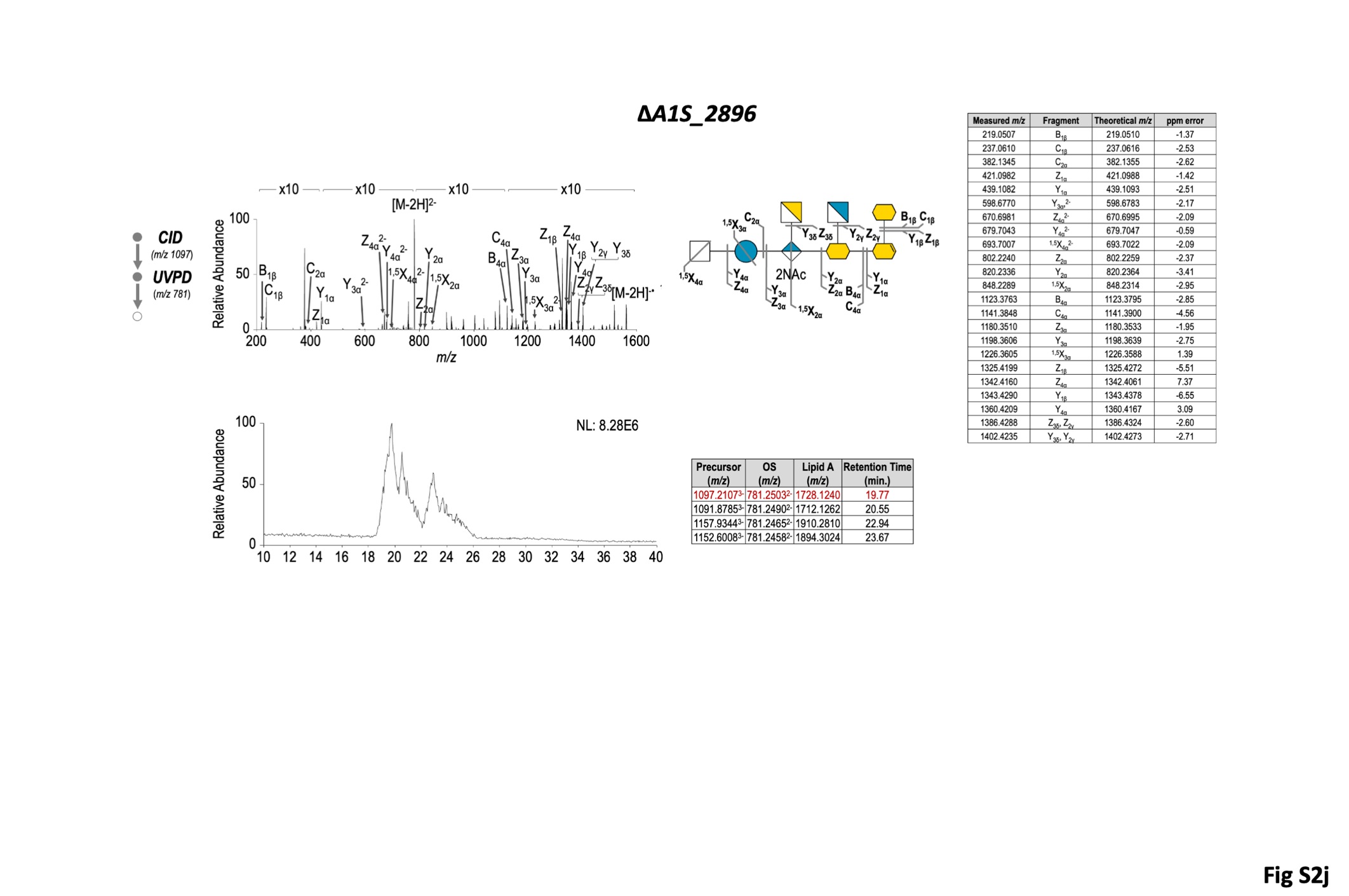


**Figure S2j: MS^3^ analysis of core oligosaccharide from *A. baumannii ∆2896.*** CID of the precursor ion of *m/z* 1097.2121 (z=3) followed by UVPD of the fragment ion of *m/z* 781.2480 (z=2). To the right of the spectrum is an oligosaccharide fragment ion map and a list of identified fragment ions with calculated ppm error. Below the spectrum is a chromatogram with a list of all identified LOS species based on CID MS^2^ spectra. Oligosaccharide fragment ion maps are depicted using the official Symbol Nomenclature for Glycans (SNFG) and Domon and Costello carbohydrate fragmentation nomenclature. H_2_O loss from Kdo upon cleavage of the glycosidic bond between the core oligosaccharide and lipid A during CID is depicted as a double bond on the Kdo symbol.


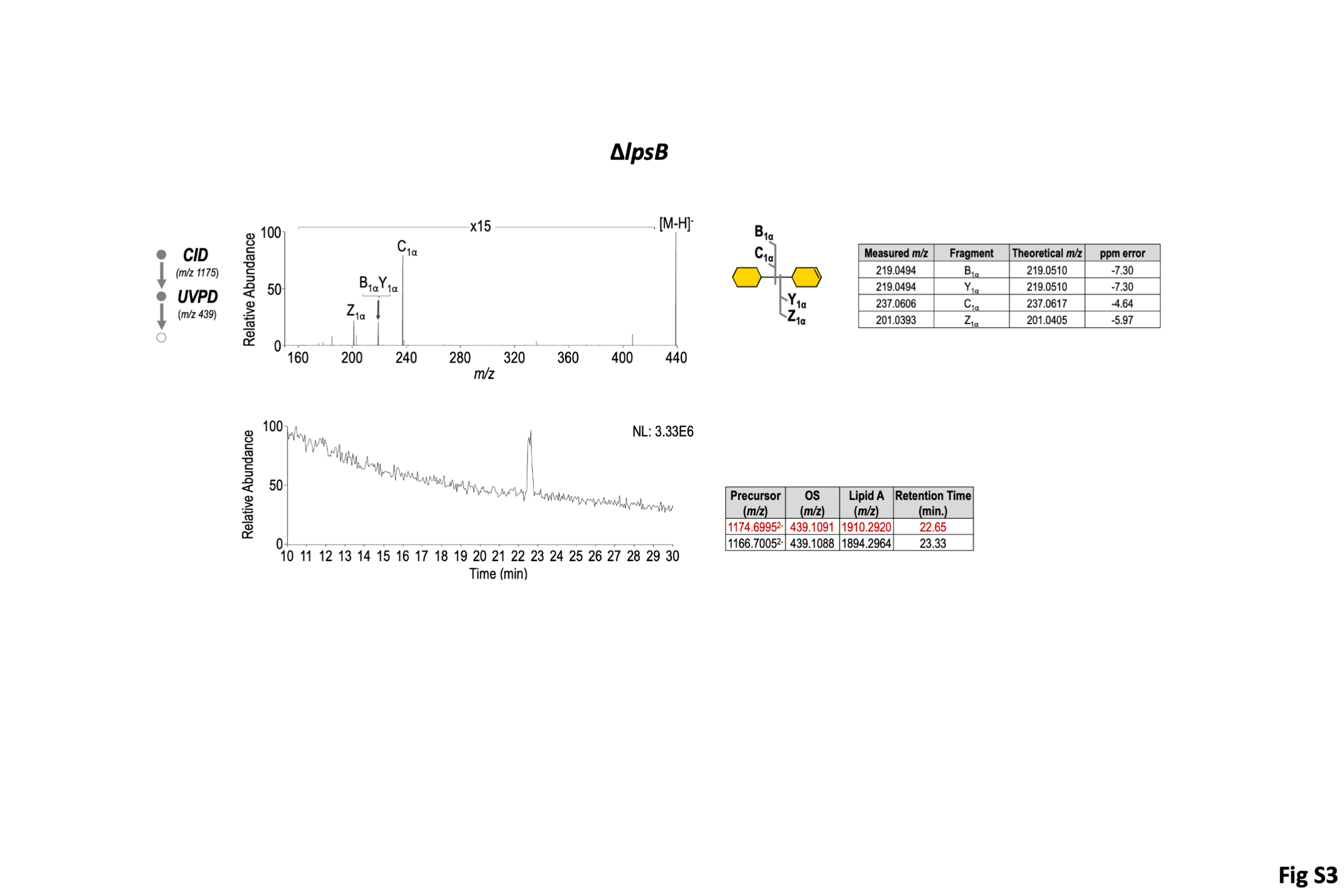


**Figure S3: MS^3^ analysis of core oligosaccharide from *A. baumannii ∆lpsB.*** CID of the precursor ion of *m/z* 1174.6995 (z=2) followed by UVPD of the fragment ion of *m/z* 439.1091 (z=1). To the right of the spectrum is an oligosaccharide fragment ion map and a list of identified fragment ions with calculated ppm error. Below the spectrum is a chromatogram with a list of all identified LOS species based on CID MS^2^ spectra. Oligosaccharide fragment ion maps are depicted using the official Symbol Nomenclature for Glycans (SNFG) and Domon and Costello carbohydrate fragmentation nomenclature. H_2_O loss from Kdo upon cleavage of the glycosidic bond between the core oligosaccharide and lipid A during CID is depicted as a double bond on the Kdo symbol.

**
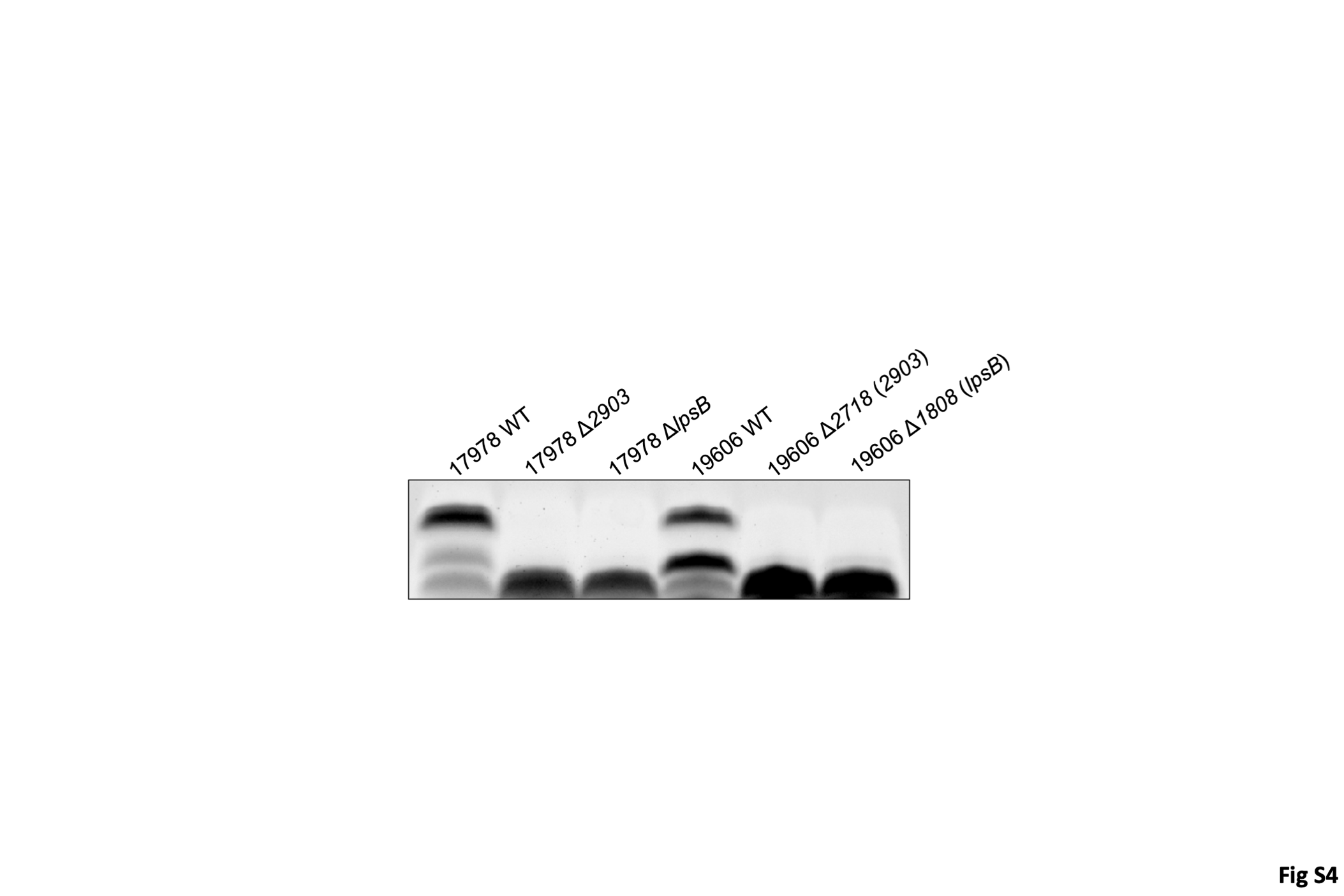
**

**Figure S4: Deletion of the *2903* and *lpsB* homologs in strain 19606 result in Kdo_2_-lipid A chemotypes.** Analysis of core oligosaccharides of the indicated strains via SDS-PAGE separation and ProQ Emerald 300 staining of proteinase K-treated cell lysates.

**
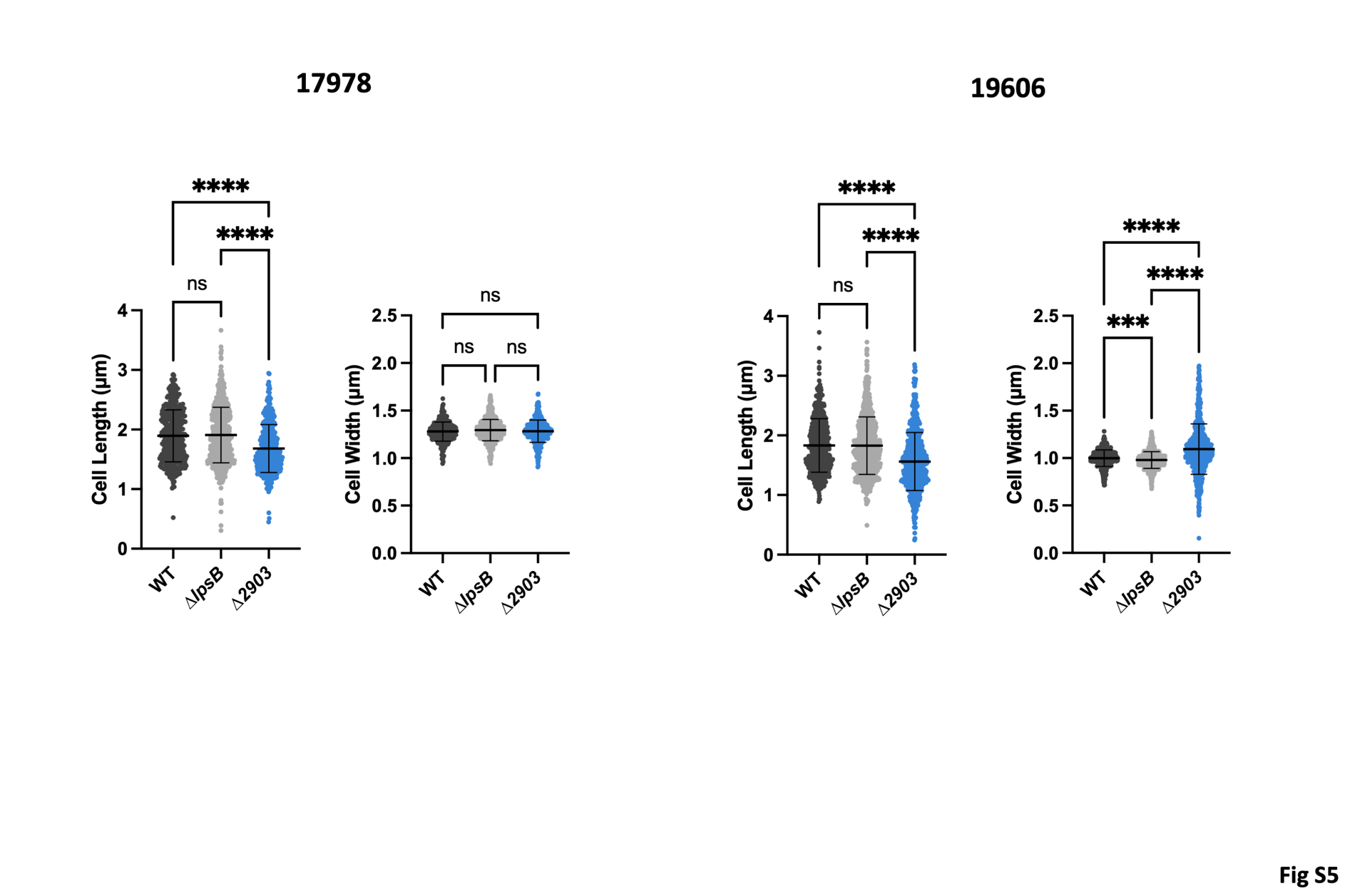
**

**Figure S5: Cell size measurements of *2903* and *lpsB* mutants.**

Cell length and width of indicated strains were measured via MicrobeJ as described in Materials and Methods. Black bar indicates mean with standard deviation. *** indicates *P =* ≤0.001; ****, *P =* ≤0.0001; ns = not significant.
